# Pathological Response Depth and Pre-Treatment Risk Stratification After Neoadjuvant Chemoradiotherapy for Rectal Cancer

**DOI:** 10.3390/medsci14060632

**Published:** 2026-10-02

**Authors:** Tibiana Negru, Loredana Gabriela Stana, Livia Stanga, Danut Dejeu, Adrian Cosmin Ilie, Stelian Pantea

**Affiliations:** 1 Department of General Surgery, Faculty of Medicine, “Victor Babeș” University of Medicine and Pharmacy, Eftimie Murgu Square 2, 300041 Timișoara, Romania; tibiana.negru@umft.ro (T.N.); pantea.stelian@umft.ro (S.P.); 2 Surgery Clinic III, “Pius Brînzeu” County Emergency Clinical Hospital, Liviu Rebreanu Boulevard 156, 300723 Timișoara, Romania; 3 Department I-Anatomy and Embryology, Faculty of Medicine, “Victor Babeș” University of Medicine and Pharmacy, Eftimie Murgu Square 2, 300041 Timișoara, Romania; loredana.stana@umft.ro; 4 Discipline of Microbiology, Faculty of Medicine, “Victor Babeș” University of Medicine and Pharmacy, 300041 Timișoara, Romania; stanga.livia@umft.ro; 5 Faculty of Medicine and Pharmacy, University of Oradea, 1 Decembrie Square 10, 410073 Oradea, Romania; 6 Surgical Oncology Department, Emergency County Hospital Oradea, Strada Gheorghe Doja 65, 410169 Oradea, Romania; 7 Department III Functional Sciences, Division of Public Health and Management, Faculty of Medicine, “Victor Babeș” University of Medicine and Pharmacy, Splaiul Tudor Vladimirescu 14, 300173 Timișoara, Romania

**Keywords:** rectal neoplasms, neoadjuvant therapy, chemoradiotherapy, neoplasm staging, treatment outcome

## Abstract

Background and Objectives: Neoadjuvant chemoradiotherapy (nCRT) is standard care for locally advanced rectal cancer, yet whether benefit derives from treatment receipt or from response depth remains debated. We examined nCRT receipt and tumour regression against outcome, and whether poor response is predictable beforehand. Materials and Methods: Eighty-nine consecutive patients undergoing resection for rectal or rectosigmoid adenocarcinoma (2020–2022; median follow-up 16 months) were analysed; outcomes were described in patients receiving nCRT (*n* = 60) and in those undergoing upfront surgery (*n* = 29), two groups whose allocation depended on stage and location and which are therefore presented as distinct clinical populations rather than as directly comparable arms. Response was graded by modified Ryan tumour regression grade. An exploratory four-item Pre-treatment Response Risk (PRR) score was derived and internally validated by bootstrap optimism correction. Comparisons used the Student *t*-test or Mann–Whitney U test and the chi-square or Fisher exact test, with logistic (maximum likelihood and Firth-penalised) and Cox regression and receiver operating characteristic analyses. Results: nCRT exposure was not statistically associated with mortality during follow-up (21.7% vs. 24.1%, *p* = 0.792), metastatic recurrence (21.7% vs. 20.7%, *p* > 0.999) or textbook outcome (35.0% vs. 34.5%, *p* > 0.999); the association with mortality remained non-significant after covariate adjustment in the whole cohort (Cox hazard ratio 0.66, 95% CI 0.24–1.83) and in a sensitivity analysis restricted to rectal tumours (Charlson-adjusted Firth odds ratio 0.52, 95% CI 0.08–3.94). The median follow-up of 16 months was too short to assess disease-free survival. Among patients who received nCRT, mortality varied with the depth of pathological response rather than with treatment receipt itself, from 0% in complete responders to 62% in poor responders (*p* < 0.001), and each one-grade worsening of the tumour regression grade multiplied the odds of death (OR 6.26, 95% CI 1.74–22.5, *p* = 0.005), although response grade, residual ypT/ypN stage and comorbidity were closely collinear. Comorbidity was associated with mortality (Charlson index, Firth-penalised OR 3.41 per point, 95% CI 1.89–6.15, *p* < 0.001) whereas nCRT was not (OR 0.38, 95% CI 0.08–1.72). In the derivation cohort, the PRR score discriminated poor response (apparent AUC 0.95, optimism-corrected 0.89) and was associated with major morbidity, which rose from 0% in the lowest to 93% in the highest risk band, and with mortality, which rose from 0% to 71% (*p* < 0.001 for each outcome). Conclusions: Within this cohort, nCRT exposure was not statistically associated with the evaluated short-term outcomes, which does not establish equivalence between strategies; depth of pathological response and host comorbidity carried the prognostic information, and routine pre-treatment variables showed promise for anticipating poor response. External validation is required before any clinical use.

## 1. Introduction

Colorectal cancer remains one of the most frequently diagnosed malignancies worldwide and a leading contributor to cancer mortality, with the rectum and rectosigmoid junction accounting for a substantial share of new cases each year [1]. The management of rectal cancer has evolved into a multidisciplinary enterprise in which imaging, systemic therapy, radiotherapy, and surgery are sequenced according to tumour stage and location [2]. Within this framework, the locally advanced tumour poses a persistent therapeutic challenge, because local control, sphincter preservation, and distant relapse must be balanced against the morbidity of intensified treatment.

Neoadjuvant chemoradiotherapy (nCRT) followed by total mesorectal excision became the reference standard after randomised evidence demonstrated improved local control and reduced toxicity when radiotherapy was delivered preoperatively rather than postoperatively [3]. Long-term follow-up of these trials confirmed durable local control benefits, although an unequivocal overall survival advantage proved more elusive [4]. Subsequent intensification strategies, including total neoadjuvant therapy, sought to increase the proportion of patients achieving a meaningful pathological response [5].

A central observation to emerge from this literature is that the outcome of neoadjuvant therapy is heterogeneous. Some tumours regress completely, whereas others progress despite identical treatment. Patients achieving a pathological complete response enjoy markedly superior disease-free and overall survival, establishing tumour regression as a powerful surrogate of biological behaviour [6]. To capture this heterogeneity reproducibly, standardised histological systems such as the modified Ryan tumour regression grade were introduced and validated across cohorts [7].

Alongside the systemic dimension, the quality of surgery itself is decisive. Completeness of the mesorectal envelope and the status of the circumferential resection margin are established determinants of local recurrence, and their assessment has been embedded in structured pathological reporting [8]. The interaction between neoadjuvant response and surgical quality has motivated organ preservation approaches in exceptional responders, further underscoring that response depth informs clinical decision-making [9]. At the same time, radiotherapy alters the surgical specimen, notably by reducing the lymph node yield available for staging [10].

The perioperative course provides an additional and often underused prognostic window. Systemic inflammatory markers measured after surgery, and composite quality metrics that aggregate morbidity, mortality, and oncological adequacy into a single patient-centred endpoint, offer pragmatic tools for risk stratification that integrate host physiology with disease biology. Whether these signals differ according to neoadjuvant exposure, and whether they track the depth of response, has been examined less systematically in unselected surgical cohorts.

Against this background, we analysed a consecutive single-centre cohort of patients operated on for rectal and rectosigmoid adenocarcinoma. Our objective was threefold: first, to describe perioperative and short-term oncological outcomes in the two treatment pathways represented in the cohort, nCRT followed by resection and upfront resection, regarding them as distinct clinical populations rather than as directly comparable arms, and to examine whether receipt of nCRT was statistically associated with these outcomes; second, to determine whether the depth of pathological response, host comorbidity, and simple perioperative biomarkers carried prognostic information beyond treatment receipt. Third, because response depth was strongly associated with outcome, we explored whether it could be anticipated from information available before any treatment was given, and derived an exploratory pre-treatment score for that purpose. We hypothesised that response depth and comorbidity would carry more prognostic information than treatment exposure alone, and that routinely measured baseline variables would carry substantial information about the response ultimately achieved.

## 2. Materials and Methods

### 2.1. Study Design and Population

This was a retrospective observational analysis of a prospectively maintained clinical surgical database comprising eighty-nine consecutive adults who underwent oncological resection for histologically confirmed rectal or rectosigmoid adenocarcinoma at a single tertiary centre between January 2020 and December 2022. All patients were discussed at a multidisciplinary tumour board, and the decision to administer neoadjuvant therapy followed contemporary stage-based recommendations, with locally advanced rectal tumours preferentially allocated to preoperative treatment. Two treatment pathways were therefore represented: nCRT followed by resection, and upfront resection. Allocation between them was determined by tumour location and by MRI-defined clinical stage, as detailed below, and not by age: no upper age limit was applied, and a patient with a stage-based indication was offered nCRT provided that the Eastern Cooperative Oncology Group performance status was 0–2, that renal and hepatic function permitted capecitabine, and that no previous pelvic irradiation had been given; comorbidity as such was not a criterion, which is reflected in the similar Charlson index of the two groups (Section 3.1). No patient was withheld from nCRT on these fitness grounds during the study period, and one patient declined it. For context, in population-based European series, roughly 79–90% of patients with clinical stage II–III rectal cancer receive some form of neoadjuvant treatment, the proportion receiving chemoradiotherapy specifically ranging from about 15 to 23% in registry populations to 68 to 87% in specialised centres [11]; the 60 of 89 resected patients (67%) treated with nCRT in the present cohort, which also includes rectosigmoid and early-stage rectal tumours, lie within this range. Eligibility required elective, curative-intent resection of a histologically confirmed adenocarcinoma of the rectum or rectosigmoid junction with complete baseline staging. Of 104 patients screened, 15 were excluded: incomplete operative or pathological documentation (*n* = 7), non-adenocarcinoma histology (*n* = 4), palliative non-resectional procedures (*n* = 2) and emergency surgery for obstruction or perforation (*n* = 2), leaving 89 patients for analysis; no patient was lost between operation and the index pathological assessment, the definitive histopathology report being issued a median of 8 working days after resection (range 5–14 days).

Tumour location was defined anatomically on rigid proctoscopy and pelvic magnetic resonance imaging (MRI): rectal cancer when the lower edge of the tumour lay within 15 cm of the anal verge (low, ≤5 cm; mid, >5–10 cm; high, >10–15 cm), and rectosigmoid cancer when the lower edge lay above 15 cm, at or above the level of the sacral promontory. Baseline clinical staging comprised pelvic MRI for all rectal tumours (clinical T and N category, distance to the mesorectal fascia and extramural venous invasion), colonoscopy with biopsy, and computed tomography of the chest, abdomen and pelvis for all patients. The indication for nCRT followed the ESMO recommendations in force during the study period [2]: MRI-defined cT3 disease with a threatened (≤1 mm) or involved mesorectal fascia, cT4 disease, clinical nodal positivity or extramural venous invasion in tumours of the low and mid rectum, and cT3–4 or node-positive disease in the high rectum. Rectosigmoid tumours were managed as colon cancer with upfront resection. Rectal tumours proceeded to upfront surgery when staged cT1–2 N0, when staged cT3 N0 with a clear mesorectal fascia in the high rectum, or when the patient declined preoperative treatment (*n* = 1).

The cohort was divided a priori into two exposure groups: patients who received neoadjuvant chemoradiotherapy before resection (*n* = 60) and patients who proceeded directly to surgery (*n* = 29). This dichotomy defined the two exposure groups. Because allocation depended on stage and location, the two groups are not clinically comparable and are presented in parallel as distinct clinical populations; wherever they are set side by side, the comparison is an observational one between non-equivalent groups, subject to confounding by indication, and is reported as such; a sensitivity analysis restricted to rectal tumours is presented in Section 3.2. Because tumour location influenced treatment allocation, the predominance of rectal tumours in the treated group was anticipated and is reported explicitly. The study was conducted in accordance with the principles of the Declaration of Helsinki and was approved as a retrospective study by the Local Commission of Ethics of the “Pius Brînzeu” County Emergency Clinical Hospital, Timișoara, on 25 June 2026 (approval no. 0944). Because of the retrospective design and the use of de-identified data, the committee waived the requirement for study-specific informed consent; all patients had provided written consent at admission for treatment and for the research use of anonymised clinical data.

### 2.2. Neoadjuvant Treatment, Surgery, Adjuvant Therapy and Follow-Up

All treated patients received long-course chemoradiotherapy: 50.4 Gy in 28 daily fractions of 1.8 Gy, delivered by a three-dimensional conformal or intensity-modulated technique to the primary tumour and the mesorectal, presacral and internal iliac nodal volumes, with concurrent oral capecitabine 825 mg/m^2^ twice daily on radiotherapy days. No patient received short-course radiotherapy or induction or consolidation chemotherapy (total neoadjuvant therapy), which had not been adopted at our institution during the study period. Radiotherapy and chemotherapy delivery were abstracted from the treatment records and expressed as the relative dose intensity (delivered divided by planned dose). Surgery was scheduled 8–12 weeks after the last fraction, following restaging MRI. All resections followed total or tumour-specific mesorectal excision principles; the procedure (anterior or low anterior resection, intersphincteric resection, abdominoperineal resection or Hartmann procedure), the approach and stoma formation were recorded. Adjuvant chemotherapy (capecitabine or capecitabine–oxaliplatin) was offered on the recommendation of the multidisciplinary board according to pathological stage.

Follow-up began on the day of surgery and comprised clinical review with carcinoembryonic antigen measurement at three-month intervals and computed tomography of the chest, abdomen and pelvis at six-month intervals, with additional imaging when symptoms arose. Vital status was ascertained from hospital records and the national population register at the data cut-off of 30 June 2023. Overall mortality was defined as death from any cause between surgery and the last follow-up, and is reported throughout as mortality during the available follow-up rather than as overall survival; cause of death was classified as postoperative (death within 90 days of surgery attributable to a complication), disease progression (death with documented metastatic recurrence) or non-cancer/undetermined. Metastatic recurrence was defined as a new distant lesion on surveillance imaging (with histological confirmation when obtained) after resection, dated to the first imaging study that identified it; patients with synchronous metastases at operation were not counted as recurrences. Patients alive at cut-off were censored at their last documented contact, and follow-up duration was summarised by the reverse Kaplan–Meier method.

### 2.3. Data Collection and Variable Definitions

For every patient, we abstracted demographic data (age, sex, body mass index), comorbidity quantified by the Charlson comorbidity index, performance status by the Eastern Cooperative Oncology Group scale, and lifestyle exposures including smoking and alcohol use. Presenting symptoms, pre-treatment laboratory values (leukocyte count, platelet count, haemoglobin, C-reactive protein) and the carcinoembryonic antigen category were recorded; in the neoadjuvant group, these were drawn at the staging visit before the first radiotherapy fraction (median 9 days before, IQR 5–14) and in the upfront group within 14 days before surgery, so that in both groups, they precede any treatment. Tumour characteristics comprised location, the maximal tumour diameter measured on baseline imaging before any treatment (Section 2.5) and, on the resection specimen, the pathological maximal diameter, histological subtype and grade, lymphovascular invasion, and the pathological tumour–node–metastasis category assigned according to the eighth edition of the American Joint Committee on Cancer staging manual [12]. In this notation, the prefix c denotes the clinical category assigned on imaging before any treatment (cT, cN, cM), the prefix *p* the category assigned on the resection specimen (pT, pN), and the prefix y a pathological category assessed after neoadjuvant therapy (ypT, ypN); thus, ypT0 denotes no residual primary tumour after nCRT, and pT2–pT3 denotes tumours invading the muscularis propria (T2) or extending beyond it into the perirectal tissue (T3). A glossary of the staging and pathological terms used in this study is provided in Appendix A (Table A3).

Pathological response to neoadjuvant therapy was graded on haematoxylin–eosin sections of the resection specimen with the modified Ryan tumour regression grade (grade 0, no viable tumour cells; grade 1, single cells or rare small groups of tumour cells; grade 2, residual tumour with predominant fibrosis; grade 3, extensive residual tumour with minimal or no regression), and a pathological complete response was defined as the absence of viable tumour in the primary and nodes (ypT0N0). Grades 0, 1–2 and 3 are referred to throughout as complete, partial and poor response; grading did not require immunohistochemistry, and none was used. Surgical quality descriptors included the completeness of the mesorectum and the circumferential margin status. Perioperative outcomes comprised the postoperative C-reactive protein and albumin, measured routinely on the morning of postoperative day 3 according to the institutional enhanced recovery protocol (from which the C-reactive protein-to-albumin ratio was derived), the change in haemoglobin and in leukocyte count from the pre-treatment value to postoperative day 3, length of stay, postoperative complications graded by the Clavien–Dindo classification [13], reintervention, and in-hospital and follow-up mortality. A composite textbook outcome was defined as the simultaneous achievement of no major complication (Clavien–Dindo grade below IIIa), no reintervention, survival, a clear resection margin, a nodal harvest of at least twelve nodes, and a non-prolonged length of stay, defined as a postoperative stay not exceeding 14 days (the 75th percentile of the cohort) [14]. Because radiotherapy reduces nodal yield, the textbook outcome was additionally computed without the nodal criterion and with a treatment-specific threshold of eight nodes after nCRT (twelve after upfront surgery), as sensitivity analyses.

Missing data were confined to lymphovascular invasion (not assessable in 11 patients, 12.4%) and carcinoembryonic antigen (not measured in 22 patients, 24.7%; 15 in the neoadjuvant and 7 in the upfront group). These variables were analysed on the available cases and denominators are given wherever they apply; no imputation was performed. All covariates of the regression models and all items of the Pre-treatment Response Risk score were complete in every patient, so that the models are complete-case analyses of the full cohort.

### 2.4. Exposure, Endpoints and Derived Indices

The principal exposure was receipt of neoadjuvant chemoradiotherapy. The co-primary endpoints were major postoperative morbidity (Clavien–Dindo grade IIIa or higher) and overall (all-cause) mortality during the available follow-up, analysed both as a proportion and as a time-to-event outcome (Section 2.6); secondary endpoints included metastatic recurrence, textbook outcome, and length of stay. To explore biological gradients within treated patients, the modified Ryan tumour regression grade was analysed both as an ordered category and, where appropriate, as a continuous trend variable.

Two derived indices were computed to capture host response and nodal disease burden. The C-reactive protein-to-albumin ratio, an established marker of the systemic inflammatory response, was calculated from the postoperative day 3 measurements [15]. Because these values were obtained at a single fixed timepoint that could precede or follow the clinical recognition of a complication, the postoperative markers are analysed as early markers associated with major morbidity rather than as predictors, and a sensitivity analysis excluded patients in whom the complication had been recognised on or before day 3. The lymph node ratio was defined as the number of metastatic nodes divided by the total number of nodes examined and was evaluated, in an exploratory analysis, as a continuous prognostic marker and, among node-positive patients, dichotomised at its median. These indices were selected because they are inexpensive, universally available, and biologically interpretable.

### 2.5. Derivation of an Exploratory Pre-Treatment Response Risk Score

Because the depth of pathological response was strongly associated with outcome, we examined whether it could be anticipated from information available before treatment. Candidate predictors were prespecified, before any outcome was examined, according to three criteria: measurement before the start of nCRT, availability without missing values in all 60 treated patients, and routine obtainability in any centre. Eight variables met these criteria: the pre-treatment C-reactive protein, haemoglobin, platelet count, leukocyte count, maximal tumour diameter, age, performance status and the Charlson comorbidity index. Each was first assessed univariably against the poor response stratum by receiver operating characteristic analysis. Histological grade and carcinoembryonic antigen, although strongly associated with response (Section 3.6), were not eligible: grade was assessed on the resection specimen and is therefore not a pre-treatment variable, and carcinoembryonic antigen was missing in 15 of 60 treated patients and available only as a category. Maximal tumour diameter was the largest dimension of the primary tumour on the baseline staging pelvic MRI (1.5 T, high-resolution T2-weighted sequences), performed a median of 12 days (IQR 7–19) before the first radiotherapy fraction and measured by the reporting radiologist as the greatest craniocaudal or axial extent according to the departmental structured report; MRI was available in all 60 treated patients and the same measurement convention was applied throughout. The pathological diameter of the resection specimen was not used in the score.

A composite Pre-treatment Response Risk (PRR) score was then constructed from four variables, one from each of four prespecified biological domains: systemic inflammation (C-reactive protein, platelet count or leukocyte count), anaemia (haemoglobin), tumour bulk (tumour diameter) and host condition (Charlson comorbidity index, age or performance status). Within the two domains containing more than one candidate, the variable with the highest univariable area under the curve was retained; this within-domain selection was data-driven and was therefore included in the internal validation described below. Each item was dichotomised at its Youden-optimal threshold and assigned one point, giving a score from zero to four; no regression weights were fitted, so that the instrument remains calculable at the bedside. Because both the thresholds and the item selection were established within this cohort, the apparent performance of the score is optimistically biased. We therefore quantified that bias by bootstrap internal validation following the optimism correction algorithm of Harrell and Steyerberg [16]. In each of 2000 bootstrap resamples (drawn with replacement, *n* = 60), the entire model-building procedure was repeated: univariable areas under the curve for all eight candidates, within-domain selection, Youden-optimal thresholds and score construction. The area under the curve of the resample-derived score was computed in the bootstrap sample and again when that score was applied to the original data; the optimism was their difference, and the optimism-corrected area under the curve was the apparent area under the curve of the original score minus the mean optimism across resamples. The frequency with which each candidate was selected and the distribution of the re-derived thresholds are reported as measures of stability. The model development component is reported in accordance with the TRIPOD statement [17] (Appendix A, Table A2), and the score is presented as exploratory model development rather than as a clinically usable instrument. For analysis, the score was additionally collapsed into three clinically interpretable bands.

### 2.6. Statistical Analysis

No a priori sample size or power calculation was performed: the study included all consecutive eligible patients treated during the study period, and its size was fixed by that period. Continuous variables were summarised as mean with standard deviation or median with interquartile range according to their distribution, which was assessed with the Shapiro–Wilk test. Between-group comparisons used the Student *t*-test when normality and homogeneity of variance (Levene test) held, the Welch correction when variances were unequal, and the Mann–Whitney U test otherwise. Categorical variables were compared with the Pearson chi-square test, applying the continuity correction for two-by-two tables, or the Fisher exact test when any expected cell count fell below five; where the continuity-corrected chi-square statistic was zero, the exact test is reported instead. Comparisons across the three response strata or the three risk bands used the Fisher–Freeman–Halton exact test. Exact binomial (Clopper–Pearson) 95% confidence intervals are given for key proportions.

Multivariable logistic regression examined associations with mortality. Covariates (age, Charlson comorbidity index, nCRT receipt, nodal positivity and distant metastasis) were selected on clinical grounds before modelling and were not changed thereafter; the Charlson index was entered as a continuous linear term, so that odds ratios are per one-point increase, and linearity was checked against a model with categorical strata. A propensity score approach (matching or inverse probability weighting) to the exposure comparison was considered and not pursued. With only 29 upfront patients, of whom 12 had rectal tumours, and with allocation determined almost deterministically by tumour location and clinical stage (Section 3.1), the two groups overlap in only a narrow region of covariate space: rectosigmoid tumours were almost never treated, and node-positive or cT3–4 tumours of the low and mid rectum were almost never operated upfront, so that the positivity assumption would be violated for most patients, a propensity model would be estimated from very few informative observations, and matching would have discarded the majority of the treated group. Covariate-adjusted regression with a small, prespecified covariate set, together with restriction to rectal tumours as a sensitivity analysis, was therefore preferred, and the exposure comparison is interpreted as descriptive. With 20 events, the events-per-variable ratio was four; in addition to maximum likelihood estimates, the model was therefore refitted with Firth’s penalised likelihood to reduce small-sample and sparse data bias, and the penalised estimates are regarded as primary. In the sensitivity analysis restricted to rectal tumours (*n* = 71, 15 deaths), the number of events permitted only a two-covariate Firth-penalised logistic model containing nCRT receipt and the Charlson index (continuous, per point); age, nodal positivity and distant metastasis, which were included in the main models, were omitted, and the addition of age as a third covariate was examined as a check on the stability of the estimate. Diagnostics comprised the Hosmer–Lemeshow test, the Brier score, variance inflation factors, Cook’s distance and delta–beta influence statistics, and a check for complete or quasi-complete separation. Time-to-event analyses used the Kaplan–Meier method with the log-rank test and a Cox proportional hazards model with the same covariates; the proportional hazards assumption was assessed with scaled Schoenfeld residuals. Within the treated group, an exploratory Firth model examined whether the tumour regression grade retained an association with mortality after adjustment for residual pathological stage (ypT3–4 and/or ypN-positive disease) and the Charlson index; a further Firth model examined metastatic recurrence in the whole cohort. Results are reported as odds or hazard ratios with 95% confidence intervals. Because the number of events was small, the term “independent predictor” is avoided and all multivariable estimates are regarded as hypothesis-generating. Associations between continuous variables were quantified with the Spearman rank correlation coefficient. The discriminatory performance of perioperative markers was assessed by receiver operating characteristic analysis, with the area under the curve estimated together with a bootstrap confidence interval (1000 resamples) and the optimal threshold identified by the Youden index; areas under the curve were compared with the DeLong test. A two-sided *p*-value below 0.05 was considered significant. Analyses were performed in Python 3.11 (Python Software Foundation, Wilmington, DE, USA) using the pandas (version 2.0), SciPy (1.11), statsmodels (0.14), scikit-learn (1.3) and lifelines (0.27) libraries; Firth-penalised models were fitted with the logistf package (version 1.26) in R 4.3.1 (R Foundation for Statistical Computing, Vienna, Austria).

## 3. Results

### 3.1. Baseline and Pathological Characteristics

Baseline demographic and clinical characteristics of the two exposure groups are presented in Table 1.

The groups were comparable in age (68.6 vs. 68.0 years, *p* = 0.773), body mass index (27.3 vs. 27.0, *p* = 0.415), Charlson index (median 6 vs. 5, *p* = 0.153), performance status (*p* = 0.152) and sex distribution (*p* > 0.999). Smoking, weight loss and presenting symptoms did not differ. The groups differed systematically, however, in tumour location and baseline disease extent: rectal tumours accounted for 98.3% of the neoadjuvant group versus 41.4% of the upfront group (*p* < 0.001), tumours in the neoadjuvant group lay lower (median 7 vs. 16 cm from the anal verge, *p* < 0.001), and cT3–4 disease (95.0% vs. 51.7%), clinical nodal positivity (76.7% vs. 31.0%) and clinical stage III–IV (80.0% vs. 31.0%) were all more frequent after neoadjuvant allocation (all *p* < 0.001). A threatened mesorectal fascia and extramural venous invasion were also more frequent in the treated group, although the differences did not reach significance among the twelve rectal tumours treated upfront. The two groups therefore differ in baseline risk and represent two distinct clinical populations, an nCRT pathway for locally advanced rectal tumours and an upfront pathway for rectosigmoid and early rectal tumours; their outcomes are presented side by side for description, and every between-group comparison that follows should be read as an observational comparison subject to confounding by indication rather than as a test of the two strategies against each other.

Neoadjuvant treatment delivery, surgical procedures, adjuvant therapy and follow-up are summarised in Table 2.

All treated patients received the same long-course regimen; no patient received total neoadjuvant therapy. Radiotherapy was completed to the planned 50.4 Gy in 90.0% and to at least 45 Gy in 95.0%, whereas capecitabine was dose-reduced, interrupted or discontinued in 23.3%, with a relative dose intensity below 80% in 15.0%. Surgery followed a median of 9 weeks after the last fraction. Abdominoperineal and intersphincteric resections were confined almost entirely to the neoadjuvant group, reflecting the lower tumour position, and adjuvant chemotherapy was given to a similar proportion of each group. Median follow-up was 16 months (IQR 10–24) after nCRT and 15 months (IQR 10–27) after upfront surgery (*p* = 0.71). Twenty patients died, three within 90 days of surgery from a postoperative complication, fifteen between three and nine months and two later; twelve deaths were attributable to disease progression and five to non-cancer or undetermined causes. Of the nineteen metastatic recurrences, twelve patients had died by the cut-off and seven were alive, so that mortality and recurrence overlap substantially and are modelled separately in Section 3.4.

Tumour and pathological characteristics of the resected specimens are summarised in Table 3.

Resected specimens after chemoradiotherapy contained smaller tumours (pathological diameter, median 3.2 vs. 4.0 cm, *p* < 0.001) and yielded fewer lymph nodes (median 12 vs. 16, *p* = 0.024). The number of positive nodes (*p* = 0.704) and the frequency of node-positive disease (33.3% vs. 20.7%, *p* = 0.327) did not differ. Grade, pT category, lymphovascular invasion, mesorectal completeness, margin involvement and adequate nodal yield were statistically comparable between groups.

**Figure 1:**
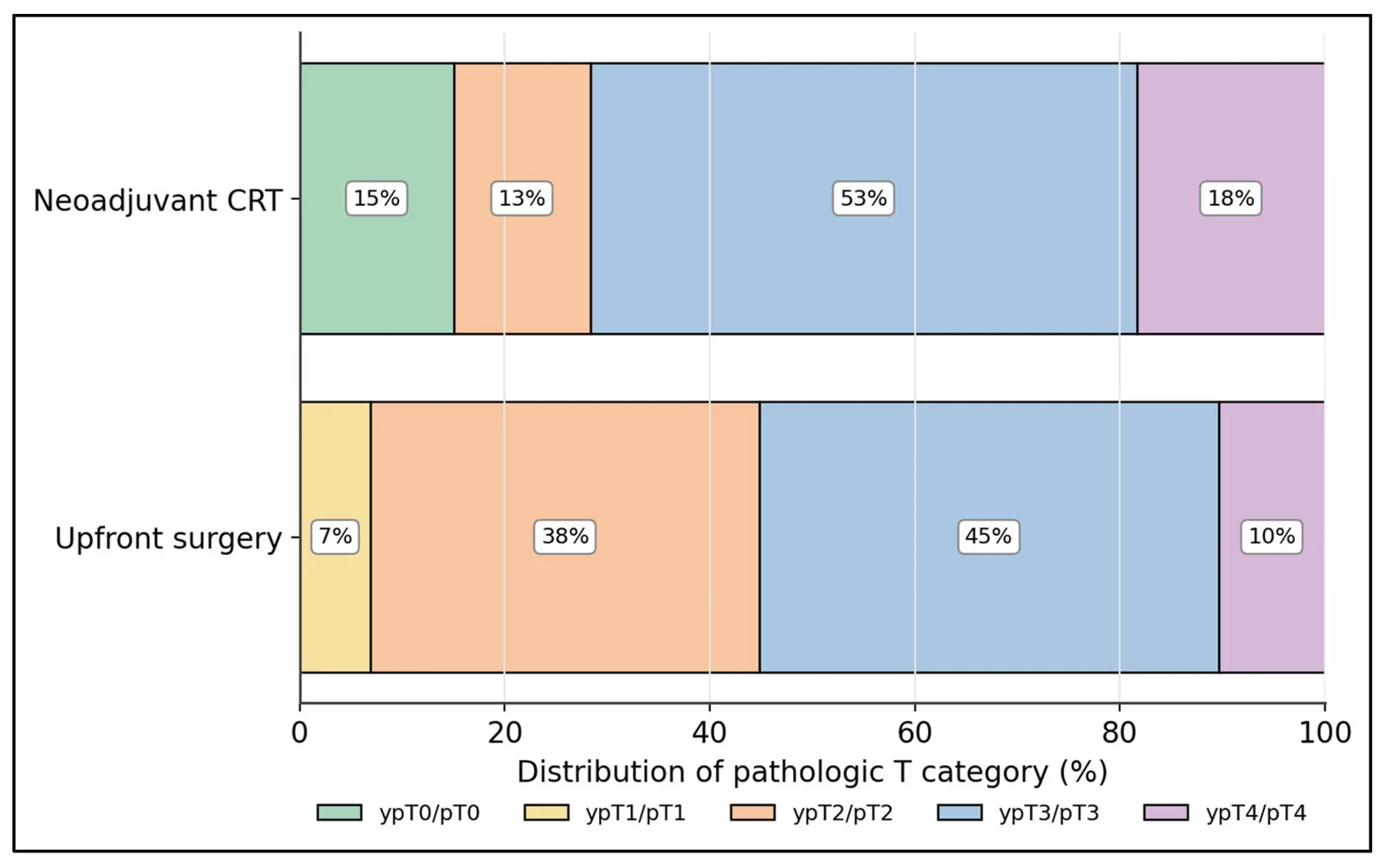
Pathological T category distribution by neoadjuvant exposure.

The distribution of the pathological T category in each exposure group is displayed in Figure 1.

A ypT0 stratum (no residual primary tumour in the resection specimen after nCRT), representing complete regression of the primary tumour, was present only after neoadjuvant chemoradiotherapy and accounted for 15% of treated specimens. The upfront group was concentrated in the pT2–pT3 range (tumours invading the muscularis propria or extending into the perirectal tissue), in which a complete response cannot occur because no preoperative treatment was given, while advanced T3–T4 disease remained the modal category in both arms.

### 3.2. Perioperative and Oncological Outcomes

Perioperative and short-term surgical outcomes are reported in Table 4.

Length of stay, antibiotic exposure, postoperative C-reactive protein, albumin and haemoglobin drop did not differ significantly between groups. Stoma formation was substantially more frequent after neoadjuvant therapy (68.3% vs. 34.5%, *p* = 0.005). Intraoperative complications, any postoperative complication, major morbidity (31.7% vs. 24.1%, *p* = 0.629), anastomotic leak and reintervention were statistically indistinguishable between the groups.

Oncological outcomes and the composite quality endpoint are presented in Table 5.

Metastatic recurrence (21.7% vs. 20.7%, *p* > 0.999), overall mortality during follow-up (21.7%, 95% CI 12–34%, vs. 24.1%, 95% CI 10–44%; *p* = 0.792), death between three and nine months (15.0% vs. 20.7%, *p* = 0.552) and textbook outcome (35.0% vs. 34.5%, *p* > 0.999) did not differ statistically between exposure groups. Kaplan–Meier estimates of survival at 12 months were 80.0% (95% CI 67.5–88.1%) after nCRT and 75.9% (95% CI 56.0–87.7%) after upfront surgery (log-rank *p* = 0.63). Kaplan–Meier estimates of freedom from metastatic recurrence at 12 months were 79.6% (95% CI 66.9–87.8%) after nCRT and 80.5% (95% CI 60.6–91.0%) after upfront surgery (log-rank *p* = 0.91); with a median follow-up of 16 months, disease-free survival beyond this early window could not be assessed, and nCRT exposure was not associated with a lower early recurrence rate in this cohort. Given the systematic baseline differences documented in Table 1, the absence of a statistically significant difference does not establish equivalence between the two strategies, and these comparisons are unadjusted observational comparisons subject to confounding by indication.

Two sensitivity analyses of the exposure comparison are presented in Table 6: restriction of the comparison to rectal tumours, and recalculation of the textbook outcome without the nodal yield criterion or with a treatment-specific nodal threshold.

Restricting the comparison to rectal tumours, which removes the imbalance in location but leaves only twelve upfront patients, did not change any conclusion: major morbidity (32.2% vs. 16.7%, *p* = 0.489), metastatic recurrence, mortality (22.0% vs. 16.7%, *p* > 0.999) and textbook outcome (35.6% vs. 41.7%, *p* = 0.748) did not differ statistically, and in a two-covariate Firth model containing nCRT receipt and the Charlson index (15 deaths among 71 patients), the Charlson-adjusted odds ratio for nCRT was 0.52 (95% CI 0.08–3.94), with the Charlson index again associated with mortality (odds ratio 3.02 per point, 95% CI 1.60–6.31, *p* = 0.001); adding age as a third covariate did not materially change the estimate for nCRT (odds ratio 0.49, 95% CI 0.07–3.81). Because the treated group had a lower nodal yield, its textbook outcome rose more than that of the upfront group when the nodal criterion was omitted (45.0% vs. 41.4%) or replaced by a treatment-specific threshold (41.7% vs. 34.5%), but neither variant produced a significant difference. The wide confidence intervals of these subgroup analyses should be noted.

### 3.3. Outcomes Stratified by Depth of Pathological Response

Within the neoadjuvant-treated cohort, ten patients showed complete regression, thirty-seven a partial response and thirteen a poor response. Outcomes across these strata are given in Table 7.

Every endpoint worsened monotonically as response deteriorated. No complete responder sustained a complication, recurrence or death. Residual pT3–T4 disease rose from 0% to 81% to 100% (*p* < 0.001) and nodal positivity from 0% to 30% to 69% (*p* = 0.001). Major morbidity increased from 0% to 19% to 92% (*p* < 0.001), mortality from 0% to 14% to 62% (*p* < 0.001), and metastatic recurrence from 0% to 16% to 54% (*p* = 0.005). Kaplan–Meier survival at 12 months was 100%, 86.5% (95% CI 70.5–94.1%) and 38.5% (95% CI 14.1–62.8%) in complete, partial and poor responders (log-rank *p* < 0.001).

**Figure 2:**
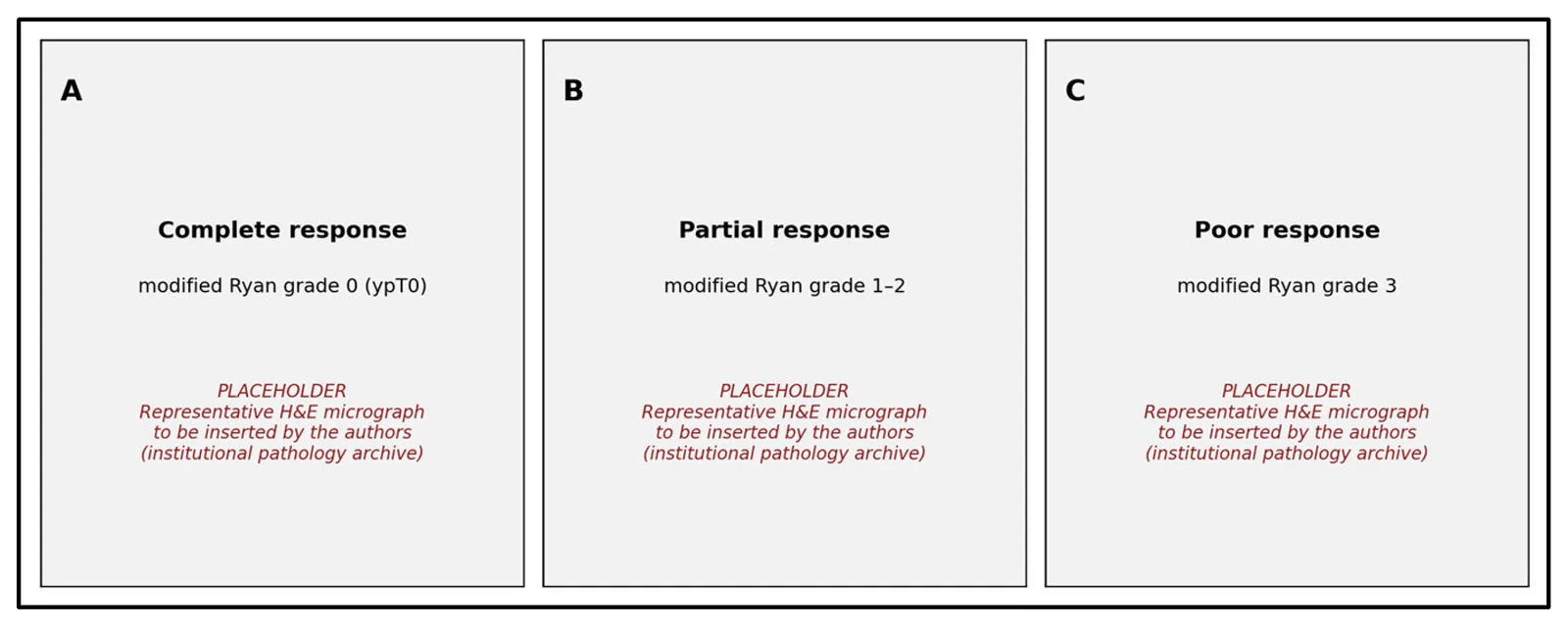
Representative haematoxylin–eosin sections of resection specimens illustrating the three pathological response strata. (**A**) Complete response (modified Ryan grade 0): fibrosis and acellular mucin in the former tumour bed without viable tumour cells (ypT0). (**B**) Partial response (grade 1–2): scattered residual tumour glands embedded in predominant fibrosis. (**C**) Poor response (grade 3): extensive residual adenocarcinoma with minimal regressive change (ypT3). Original magnification and scale bars as indicated on the panels. Response was graded on haematoxylin–eosin sections; no immunohistochemistry was used. The figure illustrates that the regression grade, rather than treatment receipt, separated the patients with an uncomplicated course from those with poor outcomes.

Representative haematoxylin–eosin sections illustrating the three response strata are shown in Figure 2.

**Figure 3:**
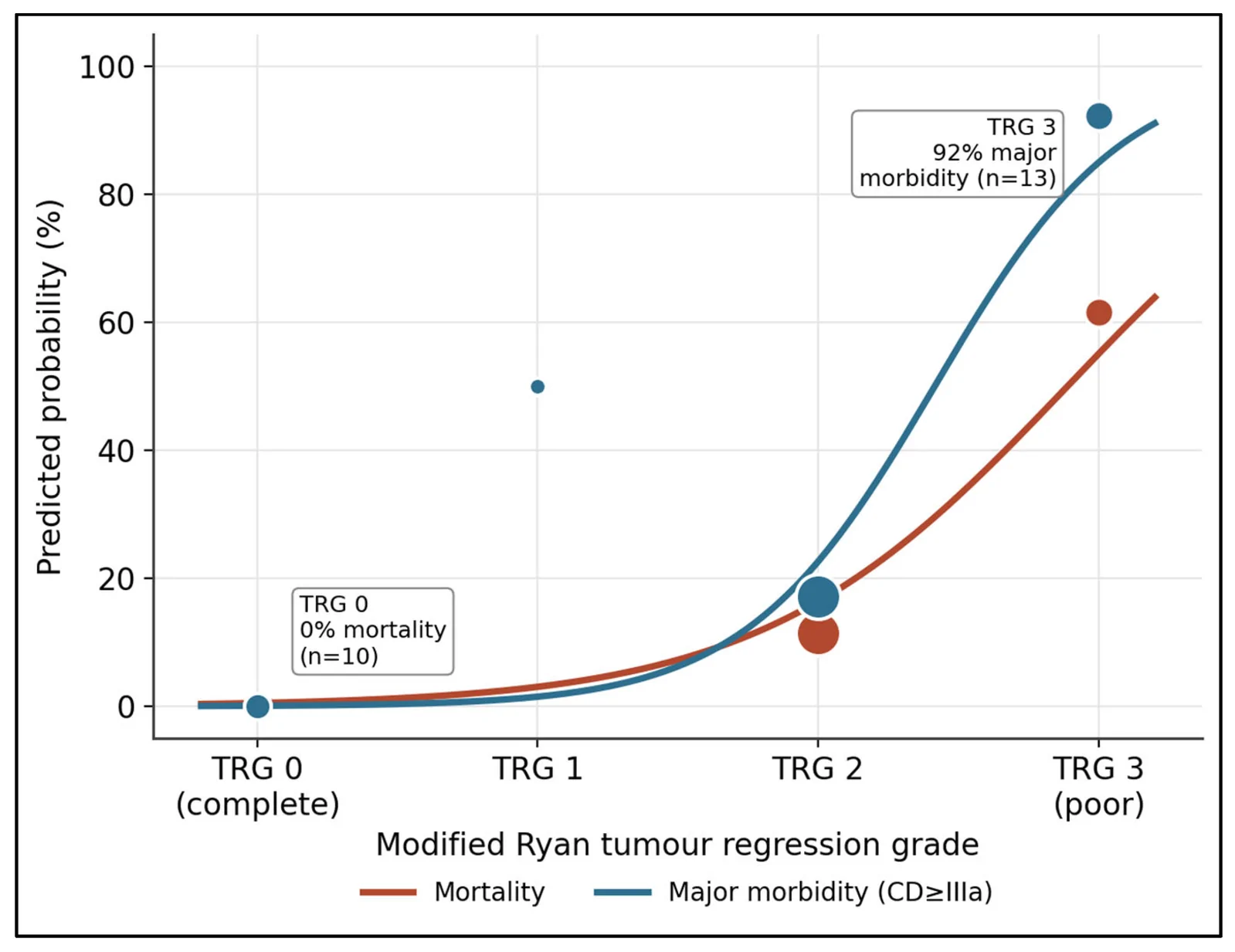
Response-graded probability of adverse outcomes. Curves are logistic model predictions; points are observed group proportions scaled to subgroup size (the area of each circle is proportional to the number of patients at that grade). The figure shows that the probability of both death and major morbidity rises steeply and monotonically with worsening regression grade, from near zero after complete response to a majority probability after poor response.

The modelled relationship between regression grade and adverse outcomes is shown in Figure 3.

Predicted probabilities of both mortality and major morbidity rose steeply and monotonically across tumour regression grades, from near zero at grade 0 (*n* = 10, no deaths) to a majority probability at grade 3 (*n* = 13; 62% mortality, 92% major morbidity). Each one-grade worsening multiplied the odds of death (odds ratio 6.26, 95% CI 1.74–22.5, *p* = 0.005; Firth-penalised odds ratio 4.90, 95% CI 1.62–18.2, *p* = 0.004).

Response grade, residual pathological stage and comorbidity were, however, closely collinear (Table 7; see also Section 3.6): every complete responder was ypT0–2 ypN0 with a Charlson index below 7, and every poor responder had residual ypT3–4 disease. The regression grade therefore cannot be separated from residual tumour burden in this dataset. In an exploratory Firth model restricted to treated patients (13 deaths; Section 3.4), the association of the tumour regression grade with mortality attenuated after adjustment for residual ypT3–4 and/or ypN-positive disease and for the Charlson index (odds ratio 2.71 per grade, 95% CI 0.78–10.4, *p* = 0.12), whereas the Charlson index remained associated (odds ratio 2.58 per point, 95% CI 1.31–5.72, *p* = 0.007). The event count does not permit a more fully adjusted model. The response gradient should therefore be read as reflecting treatment sensitivity together with residual tumour burden, rather than as an independent prognostic dimension.

### 3.4. Determinants of Recovery and Mortality

Spearman rank correlations between continuous variables and length of stay are listed in Table 8.

Length of stay correlated most strongly with the Charlson comorbidity index (ρ = +0.624, *p* < 0.001), followed by the C-reactive protein-to-albumin ratio (ρ = +0.493, *p* < 0.001) and postoperative C-reactive protein (ρ = +0.492, *p* < 0.001); postoperative albumin correlated inversely (ρ = −0.457, *p* < 0.001). Body mass index showed a weaker positive association (ρ = +0.260, *p* = 0.014), whereas haemoglobin drop and age were not significantly associated. The correlations were of similar magnitude when the analysis was restricted to the neoadjuvant group (Charlson index ρ = +0.61, C-reactive protein-to-albumin ratio +0.50, C-reactive protein +0.49, albumin −0.44; all *p* < 0.001), indicating that pooling the two exposure groups did not drive these associations.

**Figure 4:**
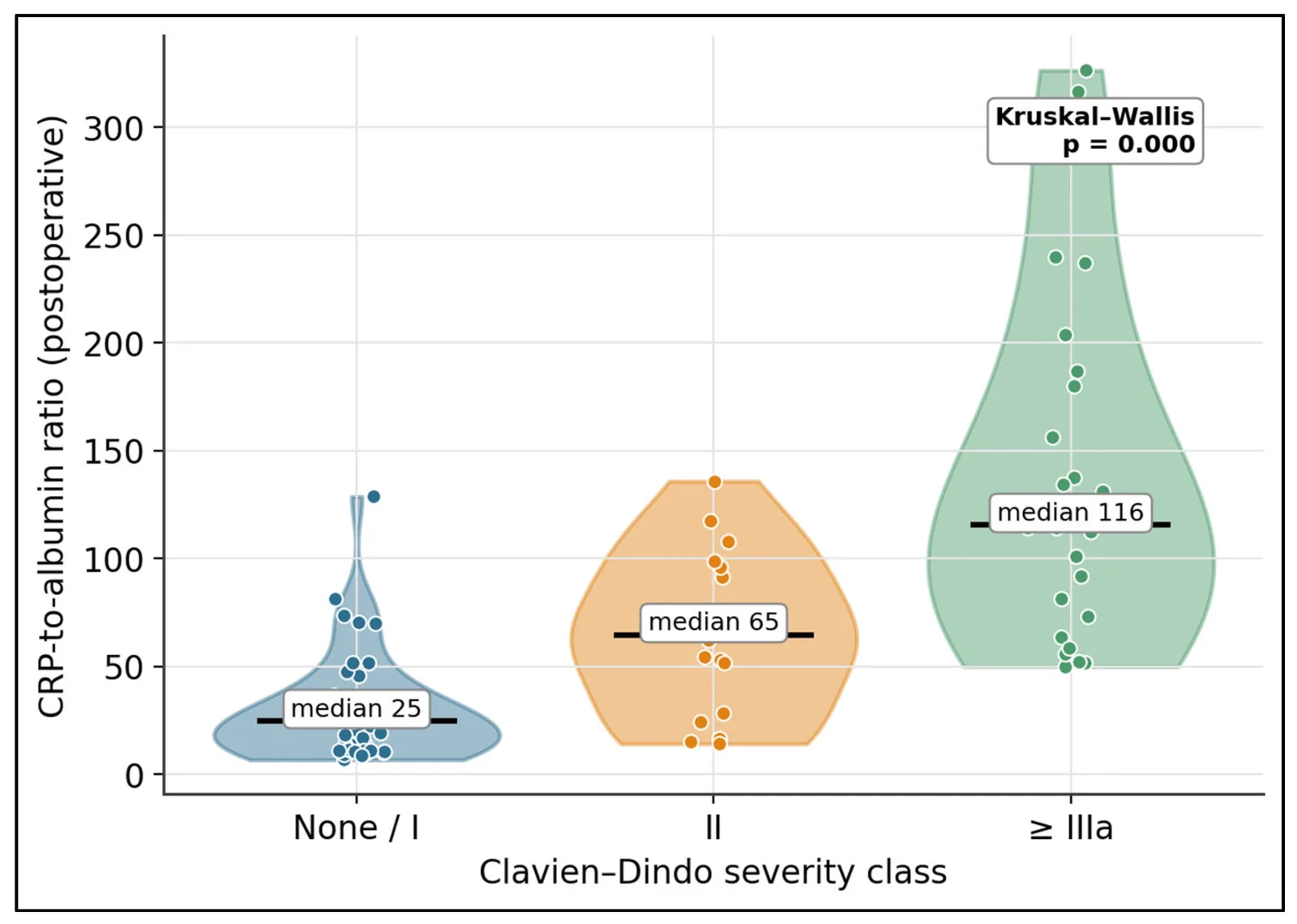
Postoperative day 3 C-reactive protein-to-albumin ratio across complication severity. Violin plots with individual observations and median markers. Colours distinguish the three severity classes (blue, none or grade I; orange, grade II; green, grade IIIa or higher) and carry no additional meaning.

The distribution of the C-reactive protein-to-albumin ratio across complication severity classes is displayed in Figure 4.

The median ratio increased across severity strata, from 24.8 in patients with no or minor complications, to 64.7 with grade II events, to 115.6 in patients with major complications (Kruskal–Wallis *p* < 0.001). Distribution width and upper-tail length also increased with severity.

Associations with overall mortality during follow-up were examined by multivariable logistic regression, with maximum likelihood and Firth-penalised estimates (Table 9).

After simultaneous adjustment, the Charlson comorbidity index was the only covariate significantly associated with death (maximum likelihood odds ratio 8.60 per point, 95% confidence interval 2.72–27.14, *p* < 0.001). On verification, the index had been coded correctly and entered as a linear term per point; the very large estimate reflects sparse data bias, because no patient with an index of 4 or less died whereas every patient with an index of 8 or more did (Table 9, footnote). The Firth-penalised estimate was 3.41 per point (95% confidence interval 1.89–6.15, *p* < 0.001), which we regard as the more reliable figure, and even this should be interpreted as imprecise. Receipt of neoadjuvant chemoradiotherapy was not associated with mortality (maximum likelihood odds ratio 0.26, 0.04–1.95, *p* = 0.191; Firth 0.38, 0.08–1.72, *p* = 0.21). Distant metastasis showed a non-significant trend (odds ratio 7.34, *p* = 0.084); age and nodal positivity were not associated.

Time-to-event analysis gave concordant results (Table 10): in the Cox model, the Charlson index (hazard ratio 1.94 per point, 95% confidence interval 1.45–2.60, *p* < 0.001) was associated with mortality, whereas nCRT receipt (hazard ratio 0.66, 0.24–1.83, *p* = 0.43), nodal positivity and age were not, and distant metastasis showed a non-significant trend (hazard ratio 3.28, 0.88–12.2, *p* = 0.077); scaled Schoenfeld residuals gave no evidence against proportional hazards (global *p* = 0.52). Because deaths and metastatic recurrences overlap substantially (twelve of the twenty deaths occurred among the nineteen patients with metastatic recurrence), metastatic recurrence was modelled separately (Table 10): pathological nodal positivity (Firth odds ratio 3.85, 95% confidence interval 1.25–12.3, *p* = 0.020) and the Charlson index (1.45 per point, 1.02–2.08, *p* = 0.039) were associated with recurrence, poorly differentiated histology showed a trend (3.21, 0.92–11.4, *p* = 0.068), and nCRT receipt was not associated (1.08, 0.30–3.95, *p* = 0.90). The timing of major complications relative to death is detailed in Appendix A (Table A1): all twenty deaths occurred in patients who had sustained a Clavien–Dindo grade IIIa or higher complication, three within 90 days of surgery as a direct consequence of the complication, fifteen between three and nine months (ten with documented metastatic progression) and two later than nine months, both with metastatic disease.

### 3.5. Perioperative Biomarkers and Nodal Disease Burden

The discrimination of the postoperative day 3 markers for major morbidity is reported in Table 11.

The C-reactive protein-to-albumin ratio showed the highest discrimination (area under the curve 0.92, 95% confidence interval 0.85–0.96), with complete sensitivity at a cut-off of 50. Postoperative C-reactive protein performed similarly (0.91), and postoperative albumin achieved high specificity (0.92 at ≤2.5 g/dL). The change in leukocyte count was weaker (0.74) and the haemoglobin drop non-discriminatory (0.46). The day 3 sample preceded clinical recognition of the complication in 21 of 26 cases (median day of diagnosis 5, IQR 4–7) but coincided with or followed it in the remaining five; these indices are therefore interpreted as concurrent early markers rather than as predictors, and discrimination was essentially unchanged when the five patients were excluded (Table 11, footnote).

**Figure 5:**
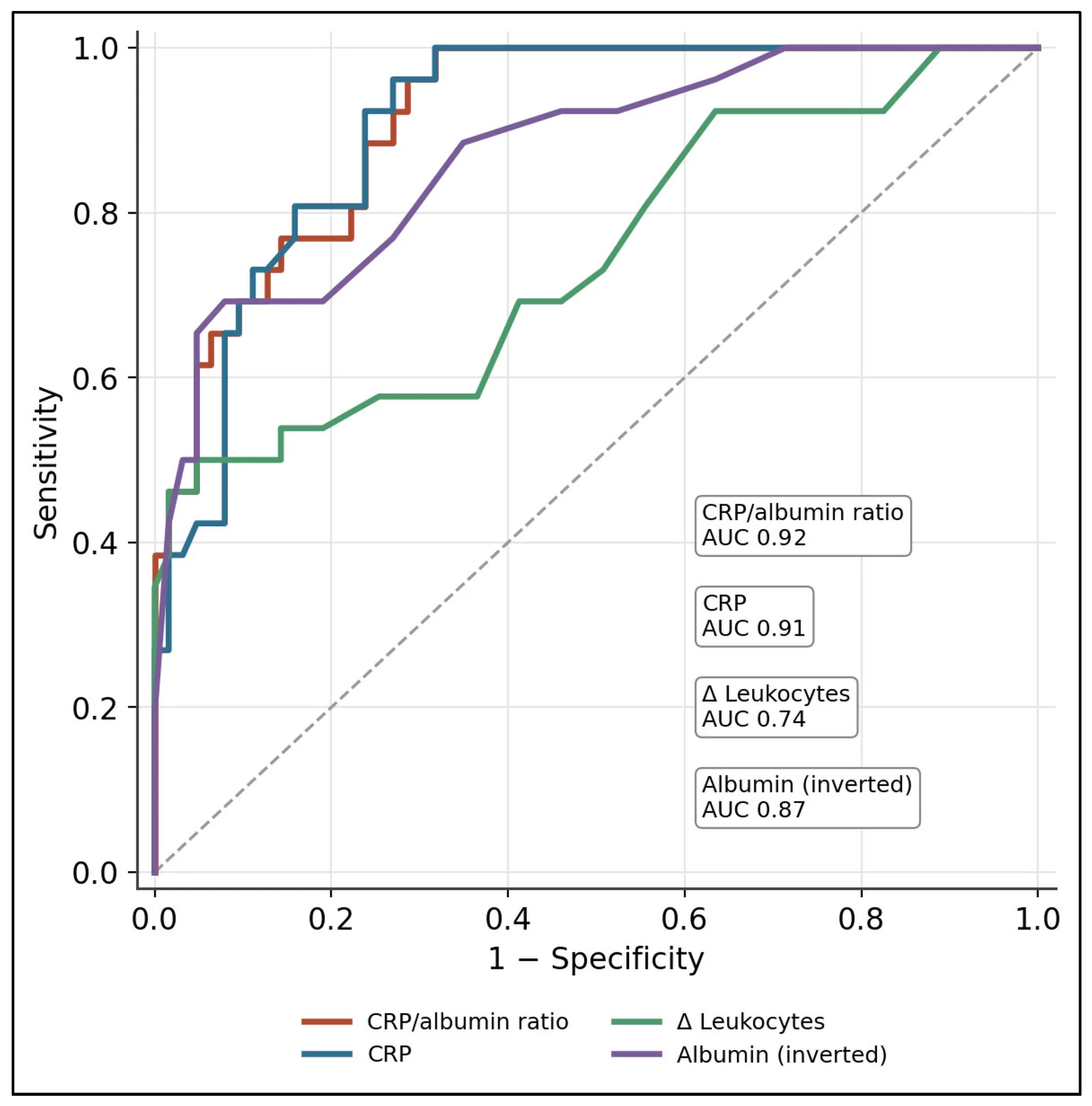
Discrimination of perioperative markers for major morbidity. Receiver operating characteristic curves with the dashed diagonal reference line of no discrimination (area under the curve 0.5). The principal message is that the C-reactive protein-to-albumin ratio and C-reactive protein discriminate major morbidity almost equally well (areas under the curve 0.92 and 0.91), whereas the change in leukocyte count adds little.

The corresponding receiver operating characteristic curves are shown in Figure 5; the markers were measured on postoperative day 3.

The curves for the C-reactive protein-to-albumin ratio and for C-reactive protein were nearly superimposed in the upper-left quadrant (areas under the curve 0.92 and 0.91), followed by albumin plotted in inverse orientation (0.87). The leukocyte change curve (0.74) lay closest to the diagonal reference line.

An exploratory analysis of the lymph node ratio relative to nodal category is summarised in Table 12.

Each 0.1 increment in the lymph node ratio more than doubled the odds of death (odds ratio 2.36, 95% confidence interval 1.58–3.54, *p* < 0.001), and the ratio showed numerically higher discrimination for mortality than the categorical pN stage (area under the curve 0.83 vs. 0.79), a difference that was not statistically significant (DeLong *p* = 0.41). Within the node-positive subgroup, differences in metastasis (83% vs. 50%, *p* = 0.110) and mortality (67% vs. 43%, *p* = 0.267) by median-dichotomised ratio did not reach significance. These analyses rest on 26 node-positive patients and are exploratory.

### 3.6. Exploratory Pre-Treatment Prediction of Pathological Response

Baseline variables recorded before any treatment differed markedly across the three response strata, as shown in Table 13.

Poor responders were older (median 74.0 vs. 62.5 years, *p* < 0.001), more comorbid (Charlson 7.0 vs. 4.0, *p* < 0.001), more inflamed (pre-treatment CRP 49.0 vs. 10.0 mg/L, *p* < 0.001), more anaemic (haemoglobin 10.8 vs. 13.3 g/dL, *p* < 0.001) and carried larger tumours on baseline MRI (4.7 vs. 2.7 cm, *p* = 0.001) than complete responders. Grade G3 histology was present in 85% of poor responders versus none of the complete responders (*p* < 0.001). Body mass index did not differ (*p* = 0.208).

Because the association between age and response could reflect poorer treatment delivery in older patients, delivery was examined by response stratum. A radiotherapy relative dose intensity below 90% occurred in 0 of 10 complete, two of 37 partial and three of 13 poor responders (*p* = 0.12), and a capecitabine relative dose intensity below 80% in 0, five and four patients respectively (*p* = 0.14). When the analysis was restricted to the 48 patients who received at least 90% of the planned radiotherapy dose and at least 80% of the planned capecitabine dose (10 complete, 30 partial and eight poor responders), median age still differed across the strata (62.5, 68.5 and 73.0 years, *p* = 0.004), indicating that reduced treatment intensity does not fully account for the age gradient, although the small numbers preclude a firm conclusion.

The composition of the exploratory Pre-treatment Response Risk score and the outcomes observed within each risk band are presented in Table 14.

No patient with a score of zero experienced a poor response, a major complication, metastatic recurrence or death (0 of 26; 95% confidence interval 0–13%). Across the three bands, poor response rose from 0% to 15% to 71%, major morbidity from 0% to 30% to 93%, and mortality from 0% to 15% to 71% (all *p* < 0.001). Textbook outcome fell from 54% to 35% to 0% (*p* = 0.001). Because these outcomes are correlated with pathological response and share the comorbidity and inflammatory components of the score, these gradients describe associations within the derivation cohort and are not an independent validation of the score.

The discriminatory performance of the score, of its individual components and of the candidate variables that were not selected is compared in Table 15.

The PRR score discriminated poor response with an area under the curve of 0.95 (95% confidence interval 0.88–0.99), which fell to 0.89 (95% confidence interval 0.79–0.95; mean optimism 0.06) after bootstrap optimism correction in which the full model-building procedure, including item selection, was repeated within each resample. Among individual components, pre-treatment CRP performed best (0.93) and tumour diameter worst (0.72); the non-selected candidates in the host and inflammation domains (ECOG 0.84, age 0.81, platelets 0.78, leukocytes 0.66) discriminated less well than the selected variable in each domain. Within the same derivation cohort, the score was also associated with major morbidity (0.95), mortality (0.92) and metastatic recurrence (0.87); because these outcomes are correlated with response and with the components of the score, these figures do not constitute independent validation.

**Figure 6:**
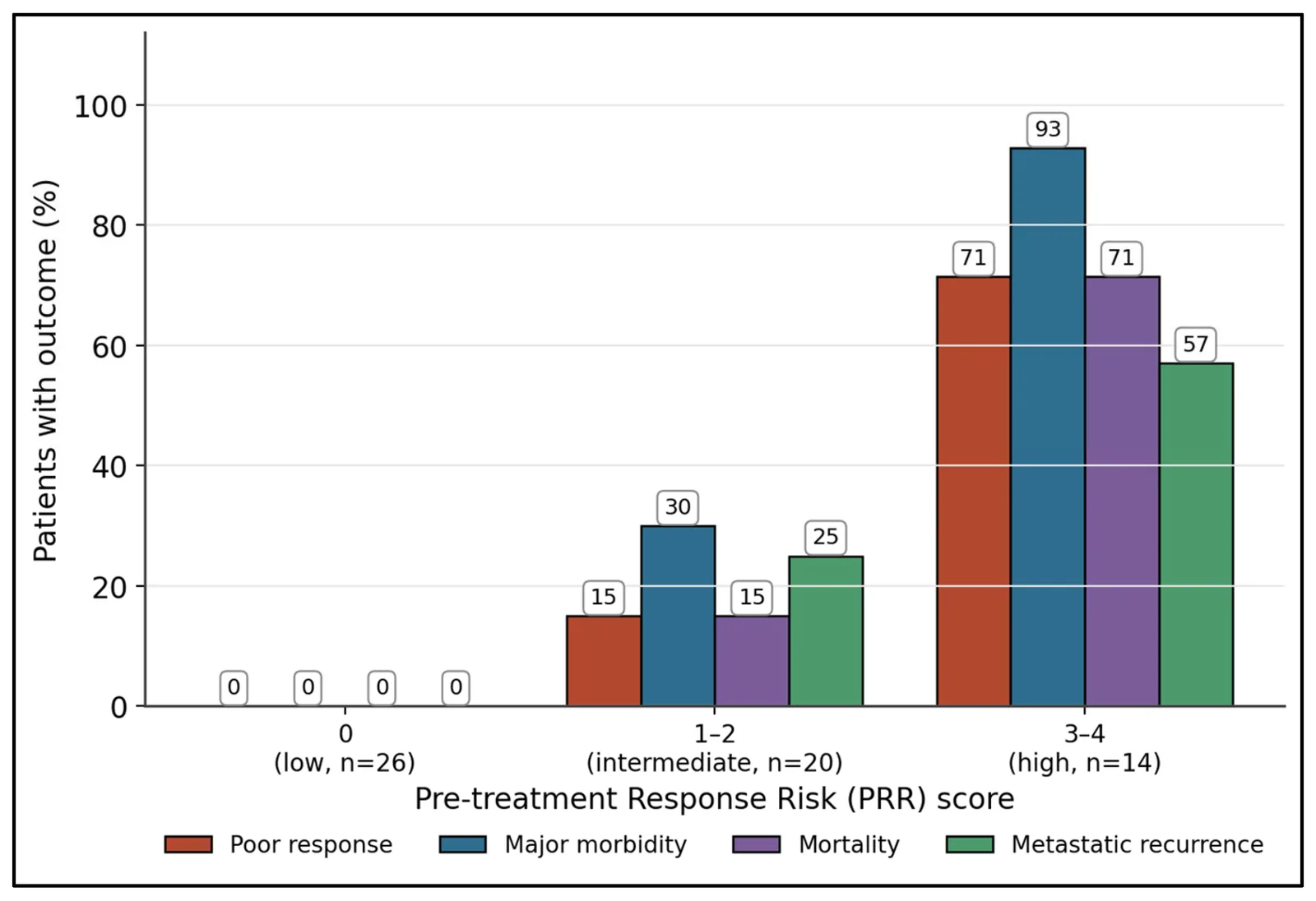
Outcomes across Pre-treatment Response Risk score bands. The figure shows that every adverse outcome increases monotonically across the three risk bands and that the lowest band contained no adverse event of any type; these are associations within the derivation cohort, not a validation of the score.

Outcome rates across the three risk bands in the derivation cohort are displayed graphically in Figure 6.

Every outcome increased monotonically across the three bands, and the low-risk band contained no events of any type. The steepest gradient was for major morbidity, which rose from 0% to 93% across the range of the score.

**Figure 7:**
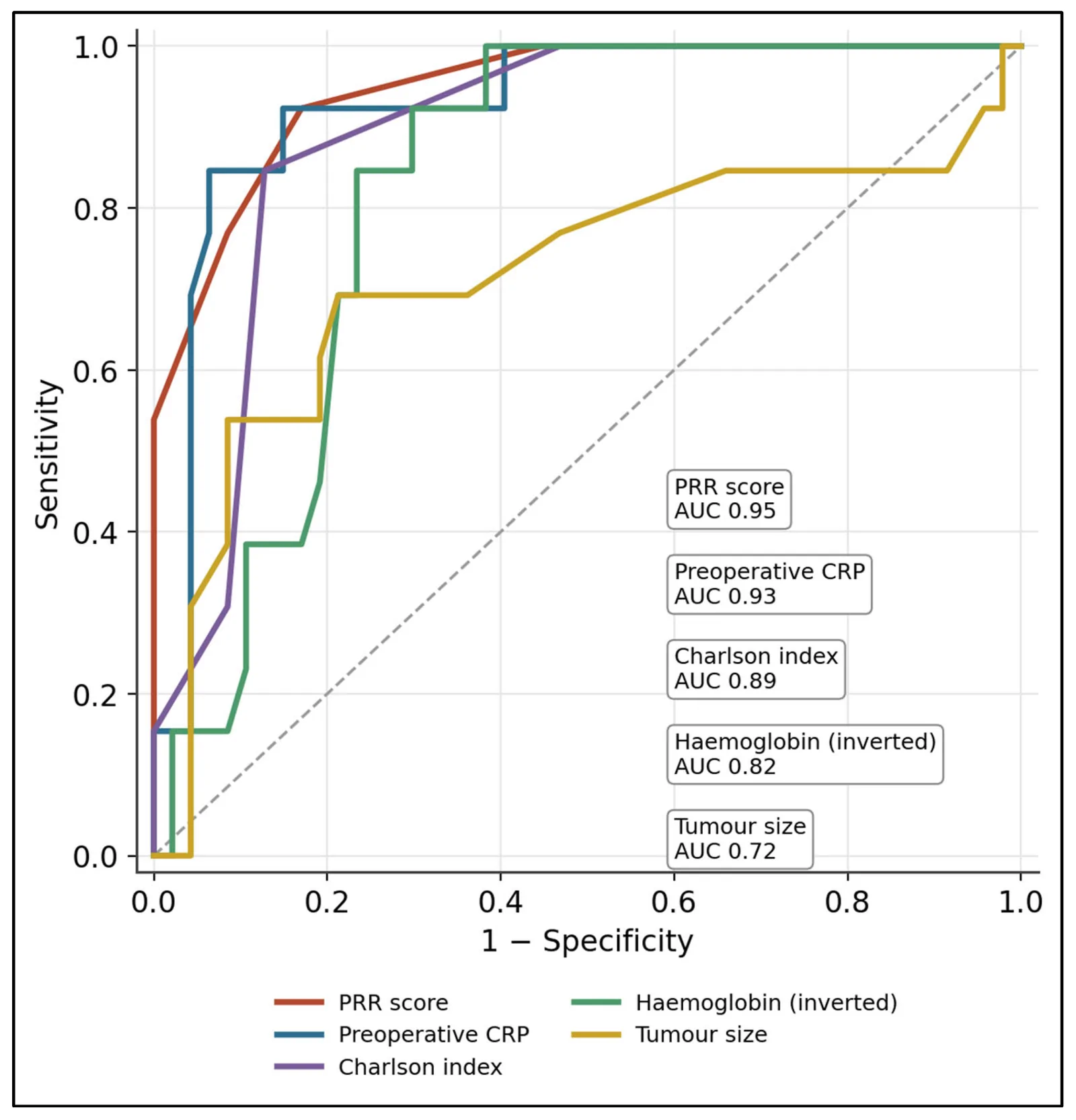
Discrimination of the Pre-treatment Response Risk score and its components for poor pathological response. The dashed diagonal line is the reference line of no discrimination (area under the curve 0.5). The composite score discriminates poor response better than any single component, of which pre-treatment C-reactive protein and the Charlson index contribute most and tumour diameter least.

Receiver operating characteristic curves for the score and its components are shown in Figure 7.

The curve for the composite score lay above those of all four individual components across most of the range of specificity. Pre-treatment CRP and the Charlson index tracked the composite most closely, whereas tumour diameter lay nearest the diagonal reference line.

## 4. Discussion

### 4.1. Analysis of Findings

The principal finding of this study is that, within this cohort, receipt of nCRT was not statistically associated with the evaluated perioperative and short-term oncological outcomes, whereas the depth of pathological response, the residual pathological stage and host comorbidity were. The two exposure groups are best regarded as two distinct clinical populations described in parallel rather than as arms of a comparison, because allocation depended on stage and location, and the treated group carried lower and more advanced disease by every clinical staging criterion. The absence of a statistically significant difference therefore neither shows that treatment receipt is unimportant nor establishes equivalence between strategies; it indicates only that nCRT exposure was not associated with these outcomes after adjustment for the covariates included in the models, and in the subgroup restricted to rectal tumours. Stratification by tumour regression grade, in contrast, showed a steep and monotonic gradient across every endpoint examined. This pattern echoes the seminal observation that complete responders enjoy exceptional survival [18] and aligns with the broader recognition that systemic inflammation and host biology, rather than treatment labels, mediate cancer outcomes [19]. The similarity of crude outcomes between the exposure groups should not be read as evidence that chemoradiotherapy is ineffective. The treated group was, by construction, enriched for lower-lying and more locally advanced rectal tumours, so that achieving parity with a lower-risk upfront population may itself represent a therapeutic effect that a crude two-group comparison is structurally unable to resolve.

Our data extend this principle by showing that response depth tracks not only oncological recurrence but also perioperative morbidity, which may suggest a shared biological substrate linking tumour radioresistance to impaired surgical recovery [20]. The gradient was large: no complete responder in this cohort experienced a complication, a recurrence or death, whereas nine of every ten poor responders sustained a major complication and nearly two-thirds died. Because tumour regression is assessed on the resection specimen, it is necessarily a retrospective marker; nevertheless, it becomes available at precisely the moment when decisions about surveillance intensity and adjuvant therapy are made, and in this cohort, it carried more information at that decision point than the treatment history itself. Response depth, residual ypT/ypN stage and comorbidity were, however, closely collinear, and in the exploratory adjusted model, the regression grade no longer carried a statistically significant association with mortality once residual stage and comorbidity were included. We therefore interpret the regression grade as a marker of treatment sensitivity that is expressed jointly with residual tumour burden, rather than as an independent prognostic dimension; separating these components will require larger cohorts.

The association of comorbidity with mortality reinforces a growing literature positioning host physiology at the centre of surgical prognostication [21]. The maximum likelihood odds ratio of 8.6 per Charlson point is implausibly large and reflects sparse data bias in a model with 20 events and quasi-complete separation at the extremes of the index; the Firth-penalised odds ratio of 3.4 per point and the Cox hazard ratio of 1.9 per point are more credible estimates, and even these are imprecise. Neoadjuvant exposure carried no statistically significant signal in any of these models. The direction of the comorbidity effect is concordant with the correlation analysis, in which comorbidity was also the strongest correlate of prolonged hospitalisation, which makes a modelling artefact less likely, although the magnitude remains uncertain. It is also notable that major morbidity had to be excluded from the mortality model because it perfectly predicted death: every patient who died had sustained a Clavien–Dindo grade IIIa or higher event. Although this separation precluded formal estimation, it is itself an informative observation (Appendix A, Table A1): most deaths occurred months after the complication, with metastatic progression documented in twelve, so that severe complications appear to have marked, and possibly accelerated, an unfavourable trajectory rather than causing death directly in most cases.

### 4.2. Comparison with the Previous Literature

The lymph node ratio, which showed numerically but not significantly higher discrimination than categorical nodal staging in our exploratory analysis, has similarly been shown to refine risk estimation in colorectal cancer, particularly where nodal harvest is compromised [22], and its stability after irradiation makes it especially attractive in the neoadjuvant setting [23]. Our observation of a reduced nodal yield after chemoradiotherapy reproduces a well-documented phenomenon and provides the mechanistic rationale for preferring a harvest-normalised metric: when the denominator shrinks, the categorical pN classification becomes progressively less stable, whereas the ratio is by construction insulated from this effect. The modest and statistically non-significant difference in discrimination that we observed is consistent with published series and should be interpreted as incremental refinement rather than replacement of conventional staging.

Our biomarker findings resonate with reports that the C-reactive protein-to-albumin ratio and related inflammatory indices are associated with postoperative complications and long-term outcome [24], and with evidence that early complications themselves compromise oncological results [25]. The near-perfect coupling of major morbidity to mortality observed here is consistent with the concept of failure-to-rescue as a determinant of surgical death [26], in which outcome depends less on whether a complication occurs than on how promptly and effectively it is recognised and treated. Viewed through that lens, the discrimination of the day 3 inflammatory indices in our cohort acquires practical significance. One caveat is that a sample drawn on a fixed day may coincide with, rather than precede, the clinical recognition of a complication. Even so, a marker that identifies every major complication, even at the cost of moderate specificity, is well matched to a rule-out role in the early postoperative period, when the cost of a missed complication greatly exceeds the cost of additional vigilance.

The absence of an association between treatment receipt and outcome in this cohort must also be set against the contemporary trend toward treatment intensification. Total neoadjuvant therapy regimens increase complete response rates relative to conventional chemoradiotherapy [27] and have improved disease-free survival in randomised trials [28]. Our findings do not contradict this evidence and, being observational, cannot clarify its mechanism; they are, however, consistent with the view that the benefit of intensification is realised through deeper pathological response. If the benefit of intensification is realised principally through deepening pathological response, then the relevant clinical question shifts from whether a patient received neoadjuvant therapy to how completely the tumour responded to it. That reframing is consistent with the response gradient documented across every endpoint in our cohort, and it may help to explain why trials that intensify treatment without stratifying for response may dilute an effect that is concentrated in a biologically defined subgroup.

### 4.3. Clinical Implications and Future Research Directions

These observations carry practical implications as the field moves toward organ preservation. Watch-and-wait strategies now spare selected exceptional responders the morbidity of resection [29], and our data support the biological premise on which such strategies rest: that deep responders constitute a distinct prognostic population rather than one end of a continuum of treatment exposure. Conversely, our poor responders represent an unmet need: their outcomes were poor in absolute terms, although a direct comparison with the upfront group is confounded by indication. Identifying these patients earlier, ideally before resection, would allow treatment to be escalated or redirected while options remain.

Emerging tools may make such early identification feasible. Circulating tumour DNA has been shown to track minimal residual disease and predict recurrence risk [30], and in mismatch-repair-deficient tumours, neoadjuvant immunotherapy has produced unprecedented complete response rates [31], suggesting that response-adapted care will increasingly be driven by molecular rather than purely histological readouts. Simple indices such as the C-reactive protein-to-albumin ratio and the lymph node ratio could complement such biomarkers at low cost, and their integration into routine assessment and enhanced recovery pathways is supported by the wider literature on the systemic inflammatory response [32,33], subject to validation.

Several priorities follow for future research. First, prospective multicentre cohorts with extended follow-up are needed to determine whether the steep early gradient we observed across response strata persists as a long-term survival difference, and whether the apparent equivalence between exposure groups conceals divergent late trajectories. Second, the diagnostic thresholds derived here require external validation before clinical adoption, ideally in cohorts large enough to permit approach-specific and stage-specific calibration. Third, the mechanistic link between tumour radioresistance and impaired surgical recovery deserves direct investigation, since a shared inflammatory or immunological substrate would open the possibility of interventions that improve both. Finally, trials of response-adapted adjuvant strategies, in which surveillance intensity and systemic therapy are calibrated to regression grade rather than to treatment history, would test the central clinical implication of this work directly.

### 4.4. Anticipating Poor Response Before Treatment

If depth of response carries the prognostic information, the natural next question is whether it can be anticipated. Our exploratory analysis suggests that part of it may be. Four routinely measured pre-treatment variables—systemic inflammation, anaemia, tumour bulk and comorbidity—identified poor responders with high apparent discrimination in the derivation cohort, and the low-risk band contained no adverse event of any kind, although with 26 patients, the upper confidence limit of that zero rate is 13%. The biological coherence of these items is notable: each indexes a different axis of the host–tumour relationship, yet they converge on the same phenotype. Elevated C-reactive protein and anaemia are recognised manifestations of the cancer-associated systemic inflammatory response, which has long been linked to treatment resistance and adverse survival [34], while tumour bulk reflects the burden of disease that radiotherapy must sterilise.

**Figure A1:**
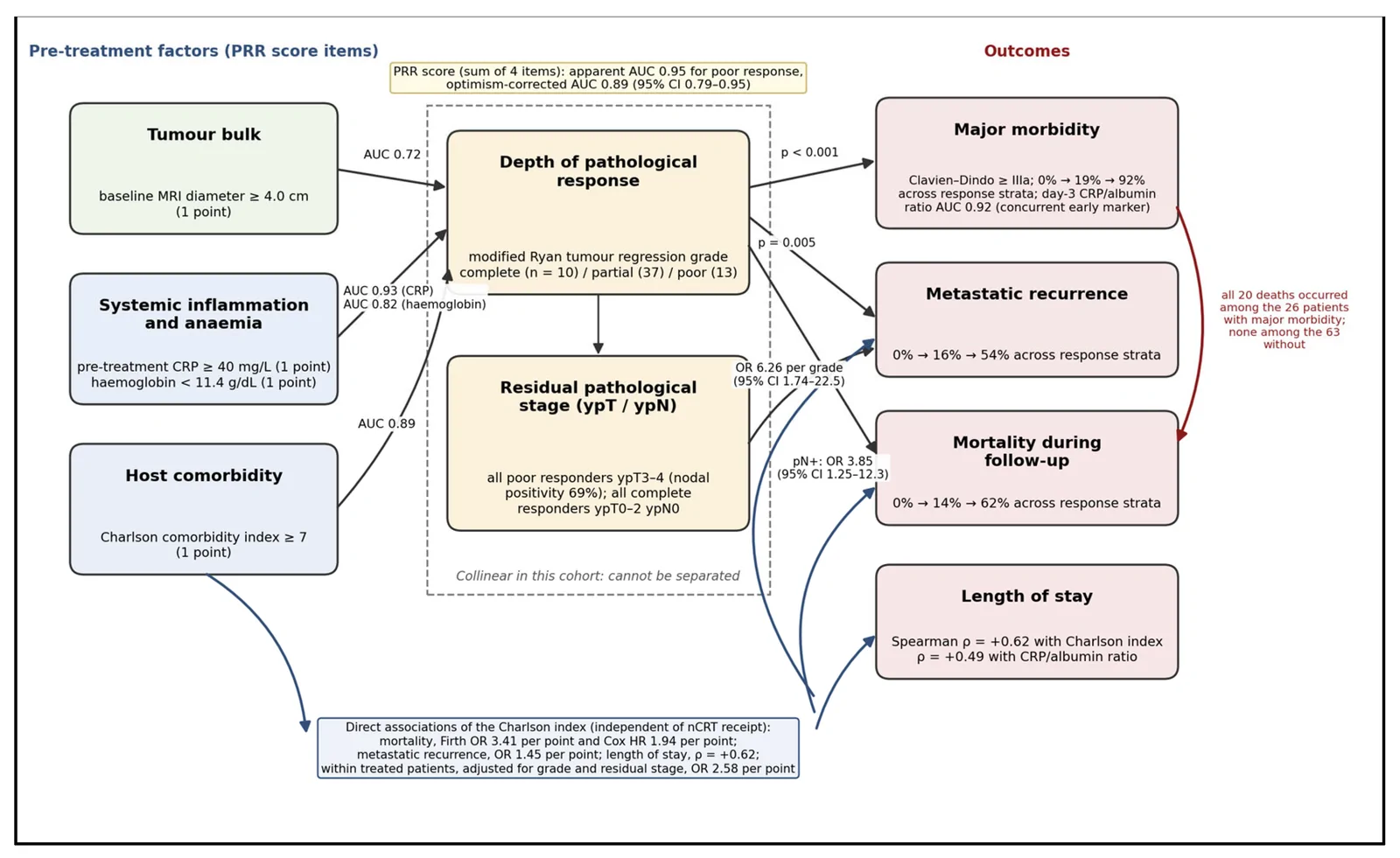
Schematic conceptual model of the interrelationships between host comorbidity, pre-treatment systemic inflammation and tumour bulk, depth of pathological response, residual pathological stage and outcome observed in this cohort. Arrows denote associations reported in Section 3.3, Section 3.4, Section 3.5 and Section 3.6 and are annotated with the corresponding estimates (areas under the curve for the prediction of poor response; odds and hazard ratios and response-stratified proportions for outcomes); the dashed frame marks the collinearity of response depth, residual ypT/ypN stage and comorbidity, which the data cannot resolve. The figure summarises observed associations and does not imply causal direction. Abbreviations: nCRT, neoadjuvant chemoradiotherapy; PRR, Pre-treatment Response Risk; CRP, C-reactive protein; MRI, magnetic resonance imaging; AUC, area under the receiver operating characteristic curve; OR, odds ratio; HR, hazard ratio; CI, confidence interval; ypT/ypN, pathological tumour/nodal category after neoadjuvant therapy; pN+, pathologically node-positive; ρ, Spearman rank correlation coefficient.

A notable structural feature of our cohort deserves comment: tumour response and host fitness co-segregated almost completely. Every complete responder had a low comorbidity burden and every poor responder a high one, so that only the partial response stratum contained both. Within that stratum, comorbidity still separated outcomes sharply, which argues that the two dimensions carry at least partly independent information. Nonetheless, this collinearity means that our data cannot disentangle whether comorbid patients respond poorly because frailty limits treatment delivery, because a shared inflammatory milieu promotes radioresistance, or because both arise from a common substrate. Disentangling these possibilities will require larger cohorts in which the two axes vary independently. The interrelationships between comorbidity, pre-treatment inflammation, response depth, residual stage and outcome discussed here, together with the corresponding estimates reported in Section 3.3, Section 3.4, Section 3.5 and Section 3.6, are summarised schematically in Appendix A (Figure A1).

Our score should be read as exploratory model development, not as a clinically usable instrument. Prediction models developed in small samples are notoriously optimistic, and internal validation by bootstrap optimism correction [16] mitigates but cannot eliminate this bias, particularly where item selection as well as threshold placement was informed by the same data. The optimism-corrected area under the curve of 0.89 was obtained by repeating the entire selection and thresholding procedure within each bootstrap sample, but internal validation cannot address the transportability of the thresholds or of the case mix, and the associations of the score with morbidity, mortality and recurrence were observed in the same cohort and are not validation. The risk of overfitting deserves to be stated explicitly. The score was built on 13 poor response events: with four items, this corresponds to roughly three events per item, far below the ten or more usually recommended for model development, and each Youden-optimal threshold was placed so as to maximise separation in these same 13 patients, as was the choice of C-reactive protein over the platelet and leukocyte counts and of the Charlson index over age and performance status. Each of these data-driven steps captures noise as well as signal; although the bootstrap re-ran all of them and estimated an optimism of only 0.06, the corrected area under the curve of 0.89 remains an estimate obtained in the derivation data and is itself imprecise (95% confidence interval 0.79–0.95), and in a new population, the thresholds in particular are likely to shift and discrimination to fall further. The literature on prognostic model development is emphatic that apparent performance in the derivation set systematically overstates external performance [35], and reporting standards require that such models be presented as provisional until validated elsewhere [17]. Comparable pre-treatment predictors of regression, including inflammatory indices and radiomic or molecular signatures, have been proposed with encouraging derivation performance but inconsistent external replication [36], a pattern that counsels humility about the figures reported here.

### 4.5. Study Limitations

Several limitations temper the interpretation of these findings. First, no a priori sample size or power calculation was performed, the sample being defined by all eligible patients treated during the study period; the retrospective, single-centre design and the modest sample of eighty-nine patients therefore constrain statistical power, particularly for subgroup and biomarker analyses, and are reflected in the wide confidence intervals of the regression models; the events-per-variable ratio of the mortality model was low, so the odds ratios, while directionally robust, should be regarded as hypothesis-generating rather than definitive; the maximum likelihood odds ratio for the Charlson index in particular is inflated by sparse data bias, which is why Firth-penalised and Cox estimates are also reported. Second, treatment allocation was not randomised, the two groups were of unequal size (60 vs. 29), and tumour location differed markedly between them, introducing confounding by indication that no statistical adjustment can fully eliminate and that also precluded a stable propensity score analysis (Section 2.6); the two groups are therefore best read as distinct clinical populations described in parallel; baseline clinical stage also differed, the exposure comparison is not adjusted for the full set of allocation determinants, and the absence of a significant difference must not be read as equivalence. A sensitivity analysis restricted to rectal tumours was consistent with the primary comparison but rests on only twelve upfront patients. Third, the follow-up window was short (median 16 months) and captured only early recurrence and mortality, precluding assessment of long-term disease-free or overall survival; mortality is therefore reported as all-cause death during the available follow-up, supplemented by Kaplan–Meier and Cox analyses, and the possibility remains that early similarity between groups masks divergent late trajectories. Fourth, the composite textbook outcome and the response strata rest on locally abstracted variables whose ascertainment, though systematic, was not externally audited, and some pathological features could not be assessed in every specimen; the nodal yield criterion of the textbook outcome also disadvantages irradiated specimens, although sensitivity analyses without it, or with a treatment-specific threshold, gave the same result. Fifth, the inflammatory and nodal indices, although promising, were derived and tested within the same cohort without external validation, so their thresholds require confirmation before clinical adoption; moreover, the postoperative markers were sampled at a single fixed timepoint (day 3) that in a fifth of cases coincided with or followed the clinical recognition of the complication, so they are markers associated with, rather than predictors of, major morbidity. Sixth, the exclusion of major morbidity from the mortality model, although statistically necessary, means that the model cannot quantify the relative contributions of complications and comorbidity to death. Seventh, and most importantly for the predictive component of this work, the Pre-treatment Response Risk score was derived and tested in the same sixty patients, with only thirteen poor response events; both its items and their thresholds were selected with knowledge of the outcome, so that even the optimism-corrected discrimination should be regarded as an upper bound on what external validation would show. The near-complete co-segregation of response depth and comorbidity further means that the score may be indexing a single underlying phenotype rather than four independent domains. Eighth, response grade, residual pathological stage and comorbidity were collinear, so the data cannot establish that the regression grade carries prognostic information independent of residual stage. These constraints notwithstanding, the consistency of the response gradient across several endpoints lends some support to the central observation.

## 5. Conclusions

In this cohort of patients operated on for rectal and rectosigmoid adenocarcinoma, receipt of neoadjuvant chemoradiotherapy was not statistically associated with perioperative or short-term oncological outcomes; because the treated and upfront groups were two distinct clinical populations allocated by stage and location, this observation is descriptive, is confounded by indication and cannot establish equivalence between strategies. The depth of pathological tumour regression, together with residual pathological stage and host comorbidity, carried the prognostic information, with deep responders having an uncomplicated course and poor responders faring worse across morbidity, recurrence and mortality during follow-up. In an exploratory analysis, an unweighted score combining pre-treatment systemic inflammation, anaemia, tumour bulk and comorbidity separated patients who went on to respond poorly from those who did not, and identified a low-risk group in which no adverse event occurred. Simple perioperative indices, namely the C-reactive protein-to-albumin ratio and the lymph node ratio, were associated with major morbidity and mortality after surgery. Taken together, these findings support giving greater weight to response depth and host condition, alongside treatment history, when estimating risk in rectal cancer. Because the predictive score was derived and tested in a single small cohort, prospective multicentre validation with extended follow-up is essential before any of these signals is allowed to influence practice.

## Figures and Tables

**Table 1:** Baseline demographic, clinical and clinical staging characteristics by neoadjuvant exposure.

Characteristic	nCRT (*n* = 60)	Upfront (*n* = 29)	*p*	Test
Age, years (mean ± SD)	68.6 ± 8.4	68.0 ± 9.9	0.773	*t*-test
Body mass index (mean ± SD)	27.3 ± 1.4	27.0 ± 1.4	0.415	*t*-test
Charlson index, median (IQR)	6.0 (5–7)	5.0 (4–6)	0.153	MWU
ECOG, median (IQR)	1 (0–2)	0 (0–1)	0.152	MWU
Sex, female/male	26/34 (43.3%/56.7%)	13/16 (44.8%/55.2%)	>0.999	Fisher
Current smoker	9/60 (15.0%)	4/29 (13.8%)	>0.999	Fisher
Weight loss	12/60 (20.0%)	5/29 (17.2%)	0.982	Chi-square
Pain at presentation	31/60 (51.7%)	11/29 (37.9%)	0.322	Chi-square
Rectal bleeding	22/60 (36.7%)	10/29 (34.5%)	>0.999	Fisher
Rectal (vs. rectosigmoid) location	59/60 (98.3%)	12/29 (41.4%)	<0.001	Fisher
Distance from anal verge, cm, median (IQR)	7 (4–10)	16 (12–18)	<0.001	MWU
Tumour height category			<0.001	Fisher
Low rectum (≤5 cm)	21/60 (35.0%)	1/29 (3.4%)		
Mid rectum (>5–10 cm)	26/60 (43.3%)	3/29 (10.3%)		
High rectum (>10–15 cm)	12/60 (20.0%)	8/29 (27.6%)		
Rectosigmoid (>15 cm)	1/60 (1.7%)	17/29 (58.6%)		
Clinical T category cT3–4	57/60 (95.0%)	15/29 (51.7%)	<0.001	Fisher
Clinical node-positive (cN1–2)	46/60 (76.7%)	9/29 (31.0%)	<0.001	Fisher
Clinical stage III–IV	48/60 (80.0%)	9/29 (31.0%)	<0.001	Fisher
Synchronous metastases (cM1)	2/60 (3.3%)	1/29 (3.4%)	>0.999	Fisher
Mesorectal fascia threatened or involved (MRI, rectal tumours)	22/59 (37.3%)	1/12 (8.3%)	0.087	Fisher
Extramural venous invasion (MRI, rectal tumours)	19/59 (32.2%)	2/12 (16.7%)	0.489	Fisher
Baseline tumour diameter on imaging, cm, median (IQR)	3.4 (2.9–4.5)	4.1 (3.5–5.4)	0.012	MWU

Clinical stage was assigned before any treatment from pelvic MRI (rectal tumours), computed tomography and colonoscopy according to the eighth edition of the AJCC manual; mesorectal fascia status and extramural venous invasion were assessed on MRI in rectal tumours only. Baseline tumour diameter is the largest dimension on staging MRI (rectal) or computed tomography (rectosigmoid). Where the continuity-corrected chi-square statistic was zero, the Fisher exact test is reported. Abbreviations: nCRT, neoadjuvant chemoradiotherapy; SD, standard deviation; IQR, interquartile range; ECOG, Eastern Cooperative Oncology Group; MWU, Mann–Whitney U test; MRI, magnetic resonance imaging; cT, clinical tumour category; cN, clinical nodal category; cM1, clinically detected distant metastasis; *p*, probability value; vs., versus.

**Table 2:** Neoadjuvant treatment delivery, surgical procedures, adjuvant therapy and follow-up by exposure group.

Variable	nCRT (*n* = 60)	Upfront (*n* = 29)	*p*	Test
Long-course CRT, 50.4 Gy/28 fractions with concurrent capecitabine	60/60 (100%)	—	—	—
Total neoadjuvant therapy or short-course radiotherapy	0/60 (0%)	—	—	—
Radiotherapy completed to 50.4 Gy	54/60 (90.0%)	—	—	—
Radiotherapy ≥ 45 Gy delivered	57/60 (95.0%)	—	—	—
Radiotherapy relative dose intensity, %, median (IQR)	100 (100–100)	—	—	—
Radiotherapy relative dose intensity < 90%	5/60 (8.3%)	—	—	—
Capecitabine relative dose intensity, %, median (IQR)	100 (85–100)	—	—	—
Capecitabine relative dose intensity < 80%	9/60 (15.0%)	—	—	—
Capecitabine dose-reduced, interrupted or discontinued	14/60 (23.3%)	—	—	—
Interval from last fraction to surgery, weeks, median (IQR; range)	9 (8–11; 6–16)	—	—	—
Surgical procedure			0.037	Fisher
Anterior or low anterior resection	33/60 (55.0%)	23/29 (79.3%)		
Abdominoperineal resection	17/60 (28.3%)	2/29 (6.9%)		
Hartmann procedure	6/60 (10.0%)	4/29 (13.8%)		
Intersphincteric resection	4/60 (6.7%)	0/29 (0%)		
Adjuvant chemotherapy	28/60 (46.7%)	14/29 (48.3%)	>0.999	Fisher
Follow-up, months, median (IQR)	16 (10–24)	15 (10–27)	0.71	MWU
Deaths during follow-up, *n*	13	7	0.792	Fisher
Within 90 days of surgery	2 (3.3%)	1 (3.4%)	>0.999	Fisher
>90 days to 9 months	9 (15.0%)	6 (20.7%)	0.552	Fisher
>9 months	2 (3.3%)	0 (0%)	>0.999	Fisher
Cause of death: postoperative/disease progression/non-cancer or undetermined	2/8/3	1/4/2	>0.999	Fisher
Time from surgery to death, months, median (IQR)	5.6 (3.8–7.4)	5.1 (3.5–6.9)	0.68	MWU
Time to detection of metastatic recurrence, months, median (IQR)	6.5 (4.5–9.0)	6.0 (4.0–8.5)	0.74	MWU
Alive with metastatic recurrence at cut-off, *n*	5	2	—	—

Relative dose intensity is the delivered divided by the planned dose. Follow-up was measured from the day of surgery to the data cut-off (30 June 2023) or last contact and summarised by the reverse Kaplan–Meier method; the earliest death occurred 0.5 months and the latest 14.8 months after surgery. Deaths within 90 days were all attributable to a postoperative complication. Abbreviations: nCRT, neoadjuvant chemoradiotherapy; CRT, chemoradiotherapy; IQR, interquartile range; MWU, Mann–Whitney U test; *p*, probability value.

**Table 3:** Tumour and pathological characteristics by neoadjuvant exposure.

Characteristic	nCRT (*n* = 60)	Upfront (*n* = 29)	*p*	Test
Pathological tumour diameter (resection specimen), cm, median (IQR)	3.2 (2.8–4.3)	4.0 (3.5–5.4)	<0.001	MWU
Nodes harvested, median (IQR)	12 (9–14)	16 (10–24)	0.024	MWU
Positive nodes, median (IQR)	0 (0–1)	0 (0–1)	0.704	MWU
Grade G3	12/60 (20.0%)	4/29 (13.8%)	0.674	Chi-square
pT3–T4 category	43/60 (71.7%)	16/29 (55.2%)	0.192	Chi-square
Node-positive (pN+)	20/60 (33.3%)	6/29 (20.7%)	0.327	Chi-square
Distant metastasis (M1)	2/60 (3.3%)	1/29 (3.4%)	>0.999	Fisher
Lymphovascular invasion	18/52 (34.6%)	9/26 (34.6%)	>0.999	Fisher
Complete mesorectum	49/60 (81.7%)	25/29 (86.2%)	0.765	Fisher
Involved margin (R1)	13/60 (21.7%)	3/29 (10.3%)	0.313	Chi-square
Adequate nodal yield (≥12)	33/60 (55.0%)	17/29 (58.6%)	0.925	Chi-square

Denominators for lymphovascular invasion exclude cases in which the feature could not be assessed (eight in the neoadjuvant and three in the upfront group). Pathological tumour diameter after nCRT refers to the residual tumour bed including fibrosis; the baseline imaging diameter is given in Table 1. Abbreviations: nCRT, neoadjuvant chemoradiotherapy; IQR, interquartile range; MWU, Mann–Whitney U test; G3, poorly differentiated histological grade; pT, pathological tumour category; pN+, pathologically node-positive; M1, distant metastasis present; R1, microscopically involved resection margin; *p*, probability value.

**Table 4:** Perioperative and short-term surgical outcomes by neoadjuvant exposure.

Outcome	nCRT (*n* = 60)	Upfront (*n* = 29)	*p*	Test
Length of stay, days, median (IQR)	11 (9–14)	9 (9–14)	0.504	MWU
Antibiotic days, median (IQR)	5 (5–7)	5 (3–7)	0.336	MWU
Postoperative day 3 CRP, mg/L, median (IQR)	169 (77–234)	88 (54–210)	0.141	MWU
Postoperative day 3 albumin, g/dL, median (IQR)	3.0 (2.5–3.3)	3.1 (2.9–3.5)	0.196	MWU
Haemoglobin drop to day 3, g/dL (mean ± SD)	1.4 ± 1.2	0.9 ± 1.1	0.074	*t*-test
Laparoscopic approach	27/60 (45.0%)	15/29 (51.7%)	0.712	Chi-square
Any stoma	41/60 (68.3%)	10/29 (34.5%)	0.005	Chi-square
Intraoperative complication	3/60 (5.0%)	1/29 (3.4%)	>0.999	Fisher
Any postoperative complication	20/60 (33.3%)	6/29 (20.7%)	0.327	Chi-square
Major morbidity (CD ≥ IIIa)	19/60 (31.7%)	7/29 (24.1%)	0.629	Chi-square
Anastomotic leak	10/60 (16.7%)	3/29 (10.3%)	0.534	Fisher
Reintervention	8/60 (13.3%)	1/29 (3.4%)	0.261	Fisher

Abbreviations: nCRT, neoadjuvant chemoradiotherapy; IQR, interquartile range; CRP, C-reactive protein; SD, standard deviation; CD, Clavien–Dindo classification; MWU, Mann–Whitney U test; *p*, probability value.

**Table 5:** Oncological outcomes and composite quality by neoadjuvant exposure.

Outcome	nCRT (*n* = 60)	Upfront (*n* = 29)	*p*	Test
Metastatic recurrence	13/60 (21.7%)	6/29 (20.7%)	>0.999	Fisher
Overall (all-cause) mortality during follow-up	13/60 (21.7%)	7/29 (24.1%)	0.792	Fisher
Death between 3 and 9 months after surgery	9/60 (15.0%)	6/29 (20.7%)	0.552	Fisher
Textbook outcome	21/60 (35.0%)	10/29 (34.5%)	>0.999	Fisher

Textbook outcome is defined in Section 2.3. Overall mortality is all-cause death during the available follow-up (median 16 months; Table 2); exact 95% confidence intervals: mortality 12–34% and 10–44%, metastatic recurrence 12–34% and 8–40%, textbook outcome 23–48% and 18–54% for the nCRT and upfront groups respectively. Where the continuity-corrected chi-square statistic was zero, the Fisher exact test is reported. Abbreviations: nCRT, neoadjuvant chemoradiotherapy; *p*, probability value.

**Table 6:** Sensitivity analyses of the exposure comparison: restriction to rectal tumours and alternative definitions of the textbook outcome.

Analysis	nCRT	Upfront	*p*	Test
Rectal tumours only (*n* = 71)	*n* = 59	*n* = 12		
Any postoperative complication	20/59 (33.9%)	4/12 (33.3%)	>0.999	Fisher
Major morbidity (CD ≥ IIIa)	19/59 (32.2%)	2/12 (16.7%)	0.489	Fisher
Anastomotic leak	10/59 (16.9%)	1/12 (8.3%)	0.676	Fisher
Reintervention	8/59 (13.6%)	0/12 (0%)	0.336	Fisher
Metastatic recurrence	13/59 (22.0%)	2/12 (16.7%)	>0.999	Fisher
Overall mortality during follow-up	13/59 (22.0%)	2/12 (16.7%)	>0.999	Fisher
Textbook outcome	21/59 (35.6%)	5/12 (41.7%)	0.748	Fisher
Mortality, Charlson-adjusted OR for nCRT (two-covariate Firth model)	0.52 (95% CI 0.08–3.94)	reference	0.55	Logistic
Textbook outcome, whole cohort (*n* = 89)	*n* = 60	*n* = 29		
Standard definition (nodal harvest ≥ 12)	21/60 (35.0%)	10/29 (34.5%)	>0.999	Fisher
Without the nodal yield criterion	27/60 (45.0%)	12/29 (41.4%)	0.822	Fisher
Treatment-specific threshold (≥8 after nCRT, ≥12 upfront)	25/60 (41.7%)	10/29 (34.5%)	0.644	Fisher

Rectal tumours are those with a lower edge within 15 cm of the anal verge. The Charlson-adjusted odds ratio derives from a Firth-penalised logistic model fitted to the 71 rectal tumour patients (15 deaths) with two covariates only, nCRT receipt and the Charlson comorbidity index entered as a continuous term per point; age, nodal positivity and distant metastasis, included in the main models, were not entered because of the number of events (Section 2.6). The nodal yield criterion was removed, or replaced by a lower threshold after nCRT, because radiotherapy reduces the number of retrievable nodes. Abbreviations: nCRT, neoadjuvant chemoradiotherapy; CD, Clavien–Dindo classification; OR, odds ratio; CI, confidence interval; *p*, probability value.

**Table 7:** Response-stratified outcomes among neoadjuvant-treated patients (*n* = 60), by modified Ryan tumour regression response.

Outcome	Complete (*n* = 10)	Partial (*n* = 37)	Poor (*n* = 13)	*p*
Pathological complete response	9 (90%)	0 (0%)	0 (0%)	<0.001
Residual pT3–T4	0 (0%)	30 (81%)	13 (100%)	<0.001
Node-positive	0 (0%)	11 (30%)	9 (69%)	0.001
Any postoperative complication	0 (0%)	12 (32%)	8 (62%)	0.006
Major morbidity (CD ≥ IIIa)	0 (0%)	7 (19%)	12 (92%)	<0.001
Overall mortality	0 (0%)	5 (14%)	8 (62%)	<0.001
Metastatic recurrence	0 (0%)	6 (16%)	7 (54%)	0.005

All comparisons by the Fisher–Freeman–Halton exact test across the three response strata; exact 95% confidence intervals for major morbidity are 0–31%, 8–35% and 64–100%, and for mortality 0–31%, 5–29% and 32–86%, in complete, partial and poor responders respectively. Small cell counts warrant cautious interpretation. The tumour regression grade was assigned by the reporting pathologist on the resection specimen (modified Ryan grade 0, complete; 1–2, partial; 3, poor). Abbreviations: pT, pathological tumour category; CD, Clavien–Dindo classification; *p*, probability value.

**Table 8:** Spearman rank correlations of continuous variables with length of stay (whole cohort).

Variable	Spearman ρ	*p*	Direction
Charlson comorbidity index	+0.624	<0.001	Longer stay
CRP-to-albumin ratio	+0.493	<0.001	Longer stay
Postoperative CRP	+0.492	<0.001	Longer stay
Postoperative albumin	−0.457	<0.001	Shorter stay
Body mass index	+0.260	0.014	Longer stay
Nodes harvested	+0.204	0.055	Longer stay (trend)
Haemoglobin drop	+0.114	0.287	Not significant
Age	+0.036	0.734	Not significant

Within the neoadjuvant group alone (*n* = 60), the corresponding coefficients were +0.61 (Charlson index), +0.50 (CRP-to-albumin ratio), +0.49 (postoperative CRP) and −0.44 (postoperative albumin), all *p* < 0.001. Postoperative markers were measured on postoperative day 3. Abbreviations: ρ, Spearman rank correlation coefficient; CRP, C-reactive protein; *p*, probability value.

**Table 9:** Multivariable logistic regression for overall mortality during follow-up: maximum likelihood and Firth-penalised estimates.

Covariate	Odds Ratio	95% CI	*p*
Maximum likelihood estimates			
Age (per year)	1.01	0.92–1.11	0.798
Charlson comorbidity index (per point)	8.60	2.72–27.14	<0.001
Neoadjuvant chemoradiotherapy	0.26	0.04–1.95	0.191
Node-positive disease	1.22	0.14–10.83	0.860
Distant metastasis	7.34	0.76–70.55	0.084
Firth-penalised estimates			
Age (per year)	1.01	0.93–1.09	0.83
Charlson comorbidity index (per point)	3.41	1.89–6.15	<0.001
Neoadjuvant chemoradiotherapy	0.38	0.08–1.72	0.21
Node-positive disease	1.30	0.22–7.55	0.77
Distant metastasis	4.92	0.62–39.0	0.13

Model fitted on *n* = 89 complete cases with 20 events (events per variable = 4); McFadden pseudo-R^2^ = 0.62 (maximum likelihood). The Charlson index was entered as a continuous linear term and odds ratios are per one-point increase; a model with categorical Charlson strata did not fit better (likelihood ratio *p* = 0.47). Diagnostics: Hosmer–Lemeshow *p* = 0.41, Brier score 0.08, all variance inflation factors < 1.6, maximum Cook’s distance 0.31, and no single observation changed the direction of any estimate on omission. Mortality by Charlson stratum was 0/30 (≤4 points), 1/22 (5), 3/17 (6), 7/11 (7) and 9/9 (≥8); this quasi-complete separation at the extremes inflates the maximum likelihood estimate, and the Firth-penalised estimates are regarded as primary. Major morbidity was excluded because it perfectly predicted death and induced numerical separation. Abbreviations: CI, confidence interval; *p*, probability value; R^2^, coefficient of determination.

**Table 10:** Time-to-event and exploratory penalised models for mortality and metastatic recurrence.

Model and Covariate	Estimate	95% CI	*p*
(a) Cox proportional hazards model for mortality, whole cohort (*n* = 89, 20 deaths)	HR		
Age (per year)	1.00	0.95–1.06	0.93
Charlson comorbidity index (per point)	1.94	1.45–2.60	<0.001
Neoadjuvant chemoradiotherapy	0.66	0.24–1.83	0.43
Node-positive disease	1.21	0.44–3.33	0.71
Distant metastasis	3.28	0.88–12.2	0.077
(b) Firth logistic model for mortality, treated patients (*n* = 60, 13 deaths)	OR		
Tumour regression grade (per grade)	2.71	0.78–10.4	0.12
Residual ypT3–4 and/or ypN-positive disease	1.92	0.16–23.5	0.58
Charlson comorbidity index (per point)	2.58	1.31–5.72	0.007
(c) Firth logistic model for metastatic recurrence, patients without synchronous metastases (*n* = 86, 19 events)	OR		
Pathological node-positive disease	3.85	1.25–12.3	0.020
Charlson comorbidity index (per point)	1.45	1.02–2.08	0.039
Grade G3 histology	3.21	0.92–11.4	0.068
Neoadjuvant chemoradiotherapy	1.08	0.30–3.95	0.90

Scaled Schoenfeld residuals gave no evidence against proportional hazards in model (a) (global *p* = 0.52). Models (b) and (c) have events-per-variable ratios of 4.3 and 4.8 respectively and are exploratory; in model (b), the tumour regression grade, residual stage and comorbidity are collinear, so the estimates are imprecise. Abbreviations: HR, hazard ratio; OR, odds ratio; CI, confidence interval; ypT/ypN, pathological tumour/nodal category after neoadjuvant therapy; *p*, probability value.

**Table 11:** Discrimination of postoperative day 3 markers for major morbidity (Clavien–Dindo ≥ IIIa).

Marker	AUC (95% CI)	Optimal Cut-Off	Sensitivity	Specificity
CRP-to-albumin ratio	0.92 (0.85–0.96)	50	1.00	0.68
Postoperative day 3 CRP (mg/L)	0.91 (0.85–0.96)	168	0.96	0.73
Postoperative day 3 albumin (g/dL)	0.87 (0.78–0.94)	≤2.5	0.69	0.92
Change in leukocytes (day 3 minus pre-treatment)	0.74 (0.62–0.86)	2.7	0.50	0.95
Haemoglobin drop (pre-treatment minus day 3)	0.46 (0.30–0.60)	—	—	—

All markers were measured on the morning of postoperative day 3. In five of the 26 major complications, the event had been clinically recognised on or before day 3; excluding these patients, the area under the curve was 0.90 (0.81–0.96) for the CRP-to-albumin ratio and 0.89 (0.80–0.95) for CRP. The markers are presented as early markers associated with major morbidity, not as predictors. Optimal cut-offs identified by the Youden index; the haemoglobin drop was non-discriminatory. Abbreviations: AUC, area under the receiver operating characteristic curve, with bootstrap confidence intervals (1000 resamples); CI, confidence interval; CRP, C-reactive protein; mg/L, milligrams per litre; g/dL, grams per decilitre.

**Table 12:** Exploratory analysis of the lymph node ratio compared with nodal category.

Analysis	Estimate	95% CI/Comparator	*p*
LNR (per + 0.1) → mortality (logistic)	OR 2.36	1.58–3.54	<0.001
LNR, discrimination for mortality (AUC)	0.83	vs. pN category 0.79	0.41 (DeLong)
LNR high vs. low → metastasis (node+ only)	83% vs. 50%	10/12 vs. 7/14	0.110
LNR high vs. low → mortality (node+ only)	67% vs. 43%	8/12 vs. 6/14	0.267

Node-positive subgroup *n* = 26 (median LNR 0.18), dichotomised at the median; all analyses are exploratory given the small number of node-positive patients. Areas under the curve were compared with the DeLong test. Abbreviations: LNR, lymph node ratio (positive nodes divided by nodes examined); OR, odds ratio; AUC, area under the receiver operating characteristic curve; pN, pathological nodal category; node+, node-positive; CI, confidence interval; *p*, probability value; vs., versus.

**Table 13:** Pre-treatment characteristics by depth of pathological response (neoadjuvant-treated patients, *n* = 60).

Pre-Treatment Variable	Complete (*n* = 10)	Partial (*n* = 37)	Poor (*n* = 13)	*p*
Age, years, median	62.5	69.0	74.0	<0.001
Charlson index, median	4.0	6.0	7.0	<0.001
ECOG, median	0	1	2	<0.001
Pre-treatment CRP, mg/L, median	10.0	20.0	49.0	<0.001
Haemoglobin, g/dL, median	13.3	11.7	10.8	<0.001
Platelets, ×10^9^/L, median	260	286	312	<0.001
Baseline MRI tumour diameter, cm, median	2.7	3.2	4.7	0.001
Grade G3 (resection specimen) †	0/10 (0%)	1/37 (3%)	11/13 (85%)	<0.001
CEA elevated	0/9 (0%)	0/26 (0%)	6/10 (60%)	<0.001
Weight loss	0/10 (0%)	6/37 (16%)	6/13 (46%)	0.015
Body mass index, median	26.7	27.5	26.7	0.208

Continuous variables compared by the Kruskal–Wallis test and categorical variables by the Fisher–Freeman–Halton exact test. Laboratory values were drawn before the first radiotherapy fraction and tumour diameter was measured on the baseline staging MRI. † Histological grade was assessed on the resection specimen and is therefore not a pre-treatment variable; it is shown for completeness but was not eligible for the score. Denominators for carcinoembryonic antigen exclude patients in whom it was not measured (*n* = 15). Abbreviations: ECOG, Eastern Cooperative Oncology Group; CRP, C-reactive protein; mg/L, milligrams per litre; g/dL, grams per decilitre; CEA, carcinoembryonic antigen; G3, poorly differentiated histological grade; *p*, probability value.

**Table 14:** Composition of the exploratory Pre-treatment Response Risk (PRR) score and outcomes by risk band in the derivation cohort (neoadjuvant-treated patients, *n* = 60).

Item (1 Point Each)	Threshold	PRR 0 (*n* = 26)	PRR 1–2 (*n* = 20)	PRR 3–4 (*n* = 14)	*p*
Pre-treatment CRP	≥40 mg/L	—	—	—	—
Haemoglobin	<11.4 g/dL	—	—	—	—
Maximal tumour diameter (baseline MRI)	≥4.0 cm	—	—	—	—
Charlson comorbidity index	≥7	—	—	—	—
Poor pathological response	outcome	0 (0%; 0–13)	3 (15%; 3–38)	10 (71%; 42–92)	<0.001
Major morbidity (CD ≥ IIIa)	outcome	0 (0%; 0–13)	6 (30%; 12–54)	13 (93%; 66–100)	<0.001
Overall mortality	outcome	0 (0%; 0–13)	3 (15%; 3–38)	10 (71%; 42–92)	<0.001
Metastatic recurrence	outcome	0 (0%; 0–13)	5 (25%; 9–49)	8 (57%; 29–82)	<0.001
Textbook outcome	outcome	14 (54%; 33–73)	7 (35%; 15–59)	0 (0%; 0–23)	0.001

The four items are summed to give a score from zero to four; thresholds were derived in this cohort by the Youden index and are therefore optimistically calibrated. Outcome rows are compared across risk bands by the Fisher–Freeman–Halton exact test; percentages are followed by exact 95% confidence intervals. Outcomes other than pathological response are associations observed in the same derivation cohort and do not constitute validation of the score. Abbreviations: PRR, Pre-treatment Response Risk; CRP, C-reactive protein; mg/L, milligrams per litre; g/dL, grams per decilitre; CD, Clavien–Dindo classification; *p*, probability value.

**Table 15:** Discrimination of the exploratory Pre-treatment Response Risk score, its components and the non-selected candidate variables for poor pathological response, with bootstrap internal validation.

Predictor	AUC (95% CI)	Optimism-Corrected AUC
PRR score (0–4)	0.95 (0.88–0.99)	0.89 (0.79–0.95)
Pre-treatment CRP (selected, inflammation domain)	0.93 (0.84–0.99)	—
Charlson comorbidity index (selected, host domain)	0.89 (0.80–0.96)	—
Haemoglobin (inverted)	0.82 (0.71–0.92)	—
Maximal tumour diameter, baseline MRI	0.72 (0.51–0.90)	—
ECOG performance status (not selected, host domain)	0.84 (0.72–0.93)	—
Age (not selected, host domain)	0.81 (0.68–0.91)	—
Platelet count (not selected, inflammation domain)	0.78 (0.64–0.89)	—
Leukocyte count (not selected, inflammation domain)	0.66 (0.49–0.81)	—
PRR score for major morbidity (same cohort, not validation)	0.95 (0.89–0.99)	—
PRR score for overall mortality (same cohort, not validation)	0.92 (0.84–0.98)	—
PRR score for metastatic recurrence (same cohort, not validation)	0.87 (0.78–0.95)	—

Confidence intervals by bootstrap resampling (2000 resamples). Optimism correction repeated the entire model-building procedure (univariable screening of all eight candidates, within-domain selection and threshold derivation) within each of 2000 bootstrap resamples and evaluated each resample-derived score in the original data; mean optimism 0.06. Selection frequencies across resamples were 94% for CRP (platelet count 5%, leukocyte count 1%) in the inflammation domain and 71% for the Charlson index (ECOG 21%, age 8%) in the host domain; haemoglobin and tumour diameter were the sole candidates in their domains. Median (IQR) re-derived thresholds: CRP 40 (32–48) mg/L, haemoglobin 11.4 (11.0–11.9) g/dL, diameter 4.0 (3.6–4.5) cm, and Charlson index 7 (6–7). The rows for major morbidity, mortality and recurrence describe associations in the same derivation cohort and are not validation of the score. Abbreviations: PRR, Pre-treatment Response Risk; AUC, area under the receiver operating characteristic curve; CI, confidence interval; CRP, C-reactive protein.

## Data Availability

The data presented in this study are available on request from the corresponding author. The data are not publicly available due to privacy and ethical restrictions.

## Appendix A

Table A1 details the timing of major postoperative complications relative to death, and Table A2 maps the model development component of this study to the TRIPOD checklist; Table A3 provides a glossary of the staging and pathological terms used in the manuscript, and Figure A1 summarises schematically the interrelationships between comorbidity, pre-treatment inflammation, pathological response, residual stage and outcome documented in Section 3.3, Section 3.4, Section 3.5 and Section 3.6.

**Table A1:** Timing of major postoperative complications (Clavien–Dindo ≥ IIIa) relative to death among the 26 affected patients.

Category	*n*	Index Major Complication	Interval from Complication to Death, Median (Range)	Cause of Death/Recurrence
Death within 90 days of surgery	3	Anastomotic leak with sepsis (2); cardiac event after reintervention (1)	18 days (9–41)	Postoperative (3); metastatic recurrence 0
Death > 90 days to 9 months	15	Anastomotic leak (6); intra-abdominal abscess requiring drainage (4); cardiopulmonary event requiring intensive care (3); fascial dehiscence requiring reoperation (2)	5.2 months (3.4–8.7)	Disease progression (10); non-cancer or undetermined (5)
Death > 9 months	2	Anastomotic leak (1); pulmonary complication requiring intensive care (1)	11.6 months (10.2–14.8)	Disease progression (2)
Alive at last follow-up	6	Anastomotic leak (4); intra-abdominal abscess requiring drainage (2)	Follow-up 19 months (12–31)	Metastatic recurrence 1, alive

All 20 deaths in the cohort occurred among the 26 patients with major morbidity; no death occurred among the 63 patients without a major complication. Complications were dated to the day of clinical diagnosis.

**Table A2:** TRIPOD checklist for the model development component (Pre-treatment Response Risk score).

TRIPOD Item	Content	Location in Manuscript
1 Title	Identification as development of a prediction score; target population and outcome	Title
2 Abstract	Objectives, design, setting, participants, sample size, predictors, outcome, analysis, results, conclusions	Abstract
3a–3b Background and objectives	Rationale for the score; development objective	Section 1
4a–4b Source of data	Retrospective single-centre cohort; study period January 2020–December 2022; follow-up to 30 June 2023	Section 2.1 and Section 2.2
5a–5c Participants	Setting, eligibility criteria, exclusions, treatments received	Section 2.1 and Section 2.2; Table 2
6a–6b Outcome	Poor pathological response (modified Ryan grade 3) assessed on the resection specimen by the reporting pathologist	Section 2.3 and Section 2.4
7a–7b Predictors	Definition and timing of pre-treatment predictors; measurement before nCRT	Section 2.3 and Section 2.5
8 Sample size	All eligible treated patients (*n* = 60, 13 events); limitation acknowledged	Section 2.1 and Section 4.5
9 Missing data	Complete data for all score items; handling of other missing data	Section 2.3
10a–10d Statistical analysis	Handling of predictors (dichotomisation at Youden thresholds), unweighted score, risk bands, bootstrap internal validation of the full procedure	Section 2.5 and Section 2.6
10e Model updating	Not applicable	—
11 Risk groups	Three bands (0, 1–2, 3–4 points)	Section 2.5; Table 14
12 Development vs. validation	Development only; no external validation	Section 2.5 and Section 4.4
13a–13b Participants	Flow of participants; characteristics	Section 2.1; Table 1 and Table 13
14a–14b Model development	Number of participants and events; univariable associations of all candidates	Section 3.6; Table 13 and Table 15
15a–15b Model specification	Full score with thresholds and points; how to calculate	Table 14
16 Model performance	Apparent and optimism-corrected discrimination; selection and threshold stability	Table 15
17 Model updating	Not applicable	—
18 Limitations	Small sample, few events, data-driven selection and thresholds, collinearity	Section 4.5
19a–19b Interpretation	Exploratory status; comparison with similar studies	Section 4.4
20 Implications	Need for external validation before any clinical use	Section 4.4 and Section 5
21 Funding	Funding statement	Funding
22 Appendix information	Appendix A	Appendix A

TRIPOD, Transparent Reporting of a multivariable prediction model for Individual Prognosis Or Diagnosis [17].

**Table A3:** Glossary of staging, pathological and statistical terms used in this study.

Term	Meaning as Used in This Study
cT, cN, cM	Clinical tumour, nodal and metastasis categories assigned before any treatment from pelvic MRI (rectal tumours), computed tomography and colonoscopy (AJCC 8th edition).
pT, pN, pM	Pathological categories assigned on the resection specimen in patients operated upfront. T1, invasion of the submucosa; T2, invasion of the muscularis propria; T3, extension beyond the muscularis propria into the perirectal tissue; T4, invasion of the visceral peritoneum or adjacent organs.
ypT, ypN	The same pathological categories assessed on a specimen resected after neoadjuvant therapy (prefix y). ypT0 denotes no residual primary tumour; ypT0N0 denotes a pathological complete response.
Modified Ryan tumour regression grade (TRG)	Histological grading of the response to neoadjuvant therapy on haematoxylin–eosin sections: grade 0, no viable tumour cells (complete response); grade 1, single cells or rare small groups; grade 2, residual tumour with predominant fibrosis (grades 1–2, partial response); grade 3, extensive residual tumour with minimal or no regression (poor response).
Locally advanced rectal cancer	Rectal cancer staged cT3–4 and/or cN1–2 on baseline MRI; the stage-based indication for neoadjuvant chemoradiotherapy (Section 2.1).
Mesorectal fascia; circumferential resection margin (R1)	The fascial envelope surrounding the rectum and its mesorectum; the surgical margin corresponding to it. A threatened (≤ 1 mm) or involved fascia on MRI is an indication for neoadjuvant treatment; R1 denotes microscopic tumour at the resection margin.
Total mesorectal excision	Removal of the rectum together with its intact mesorectal envelope; completeness of the mesorectum is a measure of surgical quality.
Clavien–Dindo classification; major morbidity	Severity grading of postoperative complications; grade IIIa or higher (requiring intervention, intensive care or resulting in death) is termed major morbidity in this study.
Charlson comorbidity index	Weighted sum of comorbid conditions (with age points) used to quantify host comorbidity; analysed per one-point increase.
ECOG performance status	Eastern Cooperative Oncology Group scale of functional status from 0 (fully active) to 4 (completely disabled).
Textbook outcome	Composite endpoint requiring, simultaneously, no major complication, no reintervention, survival, a clear resection margin, at least twelve nodes examined and a postoperative stay of 14 days or less.
Lymph node ratio (LNR)	Number of metastatic lymph nodes divided by the total number of nodes examined.
CRP-to-albumin ratio	Postoperative day 3 C-reactive protein (mg/L) divided by serum albumin (g/dL), a marker of the systemic inflammatory response.
Firth-penalised logistic regression	A logistic regression estimated with a penalty that reduces the bias of odds ratios when events are few or when a covariate separates outcomes almost completely.
Optimism correction; area under the curve (AUC)	The AUC of the receiver operating characteristic curve summarises discrimination (0.5, none; 1.0, perfect). Optimism is the amount by which apparent discrimination in the derivation data overstates expected discrimination, estimated here by bootstrap resampling.

Abbreviations: AJCC, American Joint Committee on Cancer; MRI, magnetic resonance imaging; CRP, C-reactive protein.

## References

[1] Sung H., Ferlay J., Siegel R.L., Laversanne M., Soerjomataram I., Jemal A., Bray F. (2021). Global cancer statistics 2020: GLOBOCAN estimates of incidence and mortality worldwide for 36 cancers in 185 countries. CA Cancer J. Clin..

[2] Glynne-Jones R., Wyrwicz L., Tiret E., Brown G., Rödel C., Cervantes A., Arnold D. (2017). Rectal cancer: ESMO Clinical Practice Guidelines for diagnosis, treatment and follow-up. Ann. Oncol..

[3] Sauer R., Becker H., Hohenberger W., Rödel C., Wittekind C., Fietkau R., Martus P., Tschmelitsch J., Hager E., Hess C.F. (2004). Preoperative versus postoperative chemoradiotherapy for rectal cancer. N. Engl. J. Med..

[4] Sauer R., Liersch T., Merkel S., Fietkau R., Hohenberger W., Hess C., Becker H., Raab H.-R., Villanueva M.-T., Witzigmann H. (2012). Preoperative versus postoperative chemoradiotherapy for locally advanced rectal cancer: Long-term results. J. Clin. Oncol..

[5] Cercek A., Roxburgh C.S., Strombom P., Smith J.J., Temple L.K., Nash G.M., Guillem J.G., Paty P.B., Yaeger R., Stadler Z.K. (2018). Adoption of total neoadjuvant therapy for locally advanced rectal cancer. JAMA Oncol..

[6] Maas M., Nelemans P.J., Valentini V., Das P., Rödel C., Kuo L.-J., Calvo F.A., García-Aguilar J., Glynne-Jones R., Haustermans K. (2010). Long-term outcome in patients with a pathological complete response after chemoradiation for rectal cancer: A pooled analysis. Lancet Oncol..

[7] Ryan R., Gibbons D., Hyland J.M.P., Treanor D., White A., Mulcahy H.E., O’Donoghue D.P., Moriarty M., Fennelly D., Sheahan K. (2005). Pathological response following long-course neoadjuvant chemoradiotherapy for locally advanced rectal cancer. Histopathology.

[8] Quirke P., Steele R., Monson J., Grieve R., Khanna S., Couture J., O’Callaghan C., Myint A.S., Bessell E., Thompson L.C. (2009). Effect of the plane of surgery achieved on local recurrence in rectal cancer. Lancet.

[9] van der Valk M.J.M., Hilling D.E., Bastiaannet E., Meershoek-Klein Kranenbarg E., Beets G.L., Figueiredo N.L., Habr-Gama A., Perez R.O., Renehan A.G., van de Velde C.J.H. (2018). Long-term outcomes of clinical complete responders after neoadjuvant treatment for rectal cancer (IWWD). Lancet.

[10] Mechera R., Schuster T., Rosenberg R., Speich B. (2017). Lymph node yield after rectal resection in patients treated with neoadjuvant radiotherapy: A systematic review and meta-analysis. Eur. J. Cancer.

[11] Babaei M., Jansen L., Balavarca Y., Sjövall A., Bos A., van de Velde T., Moreau M., Liberale G., Gonçalves A.F., Bento M.J. (2018). Neoadjuvant therapy in rectal cancer patients with clinical stage II to III across European countries: Variations and outcomes. Clin. Colorectal Cancer.

[12] Amin M.B., Edge S.B., Greene F.L., Byrd D.R., Brookland R.K., Washington M.K., Gershenwald J.E., Compton C.C., Hess K.R., Sullivan D.C. (2017). AJCC Cancer Staging Manual.

[13] Dindo D., Demartines N., Clavien P.A. (2004). Classification of surgical complications: A new proposal with evaluation in a cohort of 6336 patients. Ann. Surg..

[14] Kolfschoten N.E., Kievit J., Gooiker G.A., van Leersum N.J., Snijders H.S., Eddes E.H., Tollenaar R.A., Wouters M.W., Marang-van de Mheen P.J. (2013). Focusing on desired outcomes of care: The textbook outcome concept in colorectal cancer surgery. Eur. J. Surg. Oncol..

[15] Ranzani O.T., Zampieri F.G., Forte D.N., Azevedo L.C.P., Park M. (2013). C-reactive protein/albumin ratio predicts 90-day mortality of septic patients. PLoS ONE.

[16] Steyerberg E.W., Harrell F.E., Borsboom G.J., Eijkemans M.J., Vergouwe Y., Habbema J.D. (2001). Internal validation of predictive models: Efficiency of some procedures for logistic regression analysis. J. Clin. Epidemiol..

[17] Collins G.S., Reitsma J.B., Altman D.G., Moons K.G.M. (2015). Transparent reporting of a multivariable prediction model for individual prognosis or diagnosis (TRIPOD). Ann. Intern. Med..

[18] Rödel C., Martus P., Papadopoulos T., Füzesi L., Klimpfinger M., Fietkau R., Liersch T., Hohenberger W., Raab R., Sauer R. (2005). Prognostic significance of tumor regression after preoperative chemoradiotherapy for rectal cancer. J. Clin. Oncol..

[19] Roxburgh C.S.D., McMillan D.C. (2010). Role of systemic inflammatory response in predicting survival in patients with primary operable cancer. Future Oncol..

[20] Fokas E., Ströbel P., Fietkau R., Ghadimi M., Liersch T., Grabenbauer G.G., Hartmann A., Kaufmann M., Sauer R., Graeven U. (2017). Tumor regression grading after preoperative chemoradiotherapy as a prognostic factor and individual-level surrogate. J. Natl. Cancer Inst..

[21] Fagard K., Leonard S., Deschodt M., Devriendt E., Wolthuis A., Prenen H., Flamaing J., Milisen K., Wildiers H., Kenis C. (2016). The impact of frailty on postoperative outcomes in colorectal cancer surgery. J. Geriatr. Oncol..

[22] Rosenberg R., Friederichs J., Schuster T., Gertler R., Maak M., Becker K., Grebner A., Ulm K., Höfler H., Nekarda H. (2008). Prognosis of patients with colorectal cancer is associated with lymph node ratio. Ann. Surg..

[23] Ceelen W., Van Nieuwenhove Y., Pattyn P. (2010). Prognostic value of the lymph node ratio in stage III colorectal cancer: A systematic review. Ann. Surg. Oncol..

[24] Xiao Y., Zhang J., He X., Liao W., Xie Y., Sun Z., Deng H., Wang J. (2017). Preoperative C-reactive protein to albumin ratio as a prognostic indicator in colorectal cancer. Int. J. Colorectal Dis..

[25] Artinyan A., Orcutt S.T., Anaya D.A., Richardson P., Chen G.J., Berger D.H. (2015). Infectious postoperative complications decrease long-term survival in patients undergoing curative surgery for colorectal cancer. Ann. Surg..

[26] Ghaferi A.A., Birkmeyer J.D., Dimick J.B. (2009). Variation in hospital mortality associated with inpatient surgery. N. Engl. J. Med..

[27] Bahadoer R.R., Dijkstra E.A., van Etten B., Marijnen C.A.M., Putter H., Kranenbarg E.M., Roodvoets A.G.H., Nagtegaal I.D., Beets-Tan R.G.H., Blomqvist L.K. (2021). Short-course radiotherapy followed by chemotherapy before total mesorectal excision in locally advanced rectal cancer (RAPIDO). Lancet Oncol..

[28] Conroy T., Bosset J.F., Etienne P.L., Rio E., François É., Mesgouez-Nebout N., Vendrely V., Artignan X., Bouché O., Gargot D. (2021). Neoadjuvant chemotherapy with FOLFIRINOX and preoperative chemoradiotherapy for rectal cancer (PRODIGE 23). Lancet Oncol..

[29] Garcia-Aguilar J., Patil S., Gollub M.J., Kim J.K., Yuval J.B., Thompson H.M., Verheij F.S., Omer D.M., Lee M., Dunne R.F. (2022). Organ preservation in patients with rectal adenocarcinoma treated with total neoadjuvant therapy (OPRA). J. Clin. Oncol..

[30] Tie J., Cohen J.D., Wang Y., Christie M., Simons K., Lee M., Wong R., Kosmider S., Ananda S., McKendrick J. (2019). Circulating tumor DNA analyses as markers of recurrence risk and benefit of adjuvant therapy for stage III colon cancer. JAMA Oncol..

[31] Cercek A., Lumish M., Sinopoli J., Weiss J., Shia J., Lamendola-Essel M., El Dika I.H., Segal N., Shcherba M., Sugarman R. (2022). PD-1 blockade in mismatch repair-deficient, locally advanced rectal cancer. N. Engl. J. Med..

[32] Dolan R.D., McSorley S.T., Park J.H., Almasaudi A.S., Dieu L.B., McMillan D.C. (2020). The systemic inflammatory response and prognosis in cancer: A systematic review. Crit. Rev. Oncol. Hematol..

[33] Gustafsson U.O., Scott M.J., Hubner M., Nygren J., Demartines N., Francis N., Rockall T.A., Young-Fadok T.M., Hill A.G., Soop M. (2019). Guidelines for perioperative care in elective colorectal surgery: ERAS Society recommendations. World J. Surg..

[34] McMillan D.C. (2013). The systemic inflammation-based Glasgow Prognostic Score: A decade of experience in patients with cancer. Cancer Treat. Rev..

[35] Collins G.S., Ogundimu E.O., Altman D.G. (2016). Sample size considerations for the external validation of a multivariable prognostic model: A resampling study. Stat. Med..

[36] Horvat N., Veeraraghavan H., Khan M., Blazic I., Zheng J., Capanu M., Sala E., Garcia-Aguilar J., Gollub M.J., Petkovska I. (2018). MRI-based radiomics of rectal cancer: Preoperative assessment of response to neoadjuvant chemoradiation. Radiology.

